# Clinical reasoning in the emergency medical services: an integrative review

**DOI:** 10.1186/s13049-019-0646-y

**Published:** 2019-08-19

**Authors:** Ulf Andersson, Hanna Maurin Söderholm, Birgitta Wireklint Sundström, Magnus Andersson Hagiwara, Henrik Andersson

**Affiliations:** 1 Faculty of Caring Science, Work Life, and Social Welfare, University of Borås, Borås, Sweden; 2 PreHospen—Centre for Prehospital Research, University of Borås, Borås, Sweden; 3 Swedish School of Library and Information Science, University of Borås, Borås, Sweden

**Keywords:** Clinical reasoning, Decision-making, Emergency medical services, Ambulances, Clinicians

## Abstract

**Abstract:**

Clinical reasoning is the process of gathering and understanding information conducted by clinicians in the emergency medical services (EMS) so as to make informed decisions. Research on clinical reasoning spans several disciplines, but a comprehensive view of the process is lacking. To our knowledge, no review of clinical reasoning in the EMS has been conducted.

**Aim:**

The aim was to investigate the nature, deployment, and factors influencing EMS clinicians’ clinical reasoning by means of a review.

**Method:**

Data was collected through searches in electronic databases, networking among research teams, colleagues and friends, “grey literature,” and through ancestry searches. A total of 38 articles were deemed eligible for inclusion and were analyzed using descriptive thematic analysis. The analysis resulted in an overarching finding - namely, the importance for EMS clinicians to adjust for perceived control in unpredictable situations. Within this finding, 3 themes emerged in terms of EMS clinicians’ clinical reasoning: (1) maintaining a holistic view of the patient; (2) keeping an open mind; and (3) improving through criticism. Seven subthemes subsequently emerged from these three themes.

**Results:**

This review showed that EMS clinicians’ clinical reasoning begins with the information that they are given about a patient. Based on this information, clinicians calculate the best route to the patient and which equipment to use, and they also assess potential risks. They need to be constantly aware of what is happening on the scene and with the patient and strive to control the situation. This striving also enables EMS clinicians to work safely and effectively in relation to the patient, their relatives, other clinicians, associated organizations, and the wider community. A lack of contextually appropriate guidelines results in the need for creativity and forces EMS clinicians to use “workarounds” to solve issues beyond the scope of the guidelines available. In addition, they often lack organizational support and fear repercussions such as litigation, unemployment, or blame by their EMS or healthcare organization or by patients and relatives.

**Conclusion:**

Clinical reasoning is influenced by several factors. Further research is needed to determine which influencing factors can be addressed through interventions to minimize their impact on patient outcomes.

**Electronic supplementary material:**

The online version of this article (10.1186/s13049-019-0646-y) contains supplementary material, which is available to authorized users.

## Introduction

The majority of adverse events in a healthcare system can be directly associated with clinical reasoning [1–6]. Clinical reasoning is the process of collecting, evaluating, and using available information in order to make decisions. In patient encounters, clinicians in the emergency medical services (EMS) use clinical reasoning, to make assessments and decisions about how to proceed regarding the medical, care, and existential needs of patients with manifested or perceived physical, psychological, or existential discomfort [7–9]. It is not possible to separate assessment and decision-making in the clinical reasoning process; rather, they are continuous and intertwined sub-processes, dependent of each other [10, 11].

The contexts in which EMS clinicians use clinical reasoning vary. They may begin advanced medical treatment on-site or during transportation, make decisions about non-conveyance, or direct the patient to a suitable level of care [12–14]. These decisions depend on existing variations in organizational structures and the many different competencies of actors in international EMS systems [15–17]. These actors include emergency technicians, paramedics, registered nurses, specialized nurses, and physicians.

Previous research on clinical reasoning spans several research disciplines, and there does not seem to be a unified view of the content and mechanisms of the clinical reasoning process [10, 11, 18]. Studies in the context of the EMS mostly cover the accuracy of specific diagnoses (i.e. the outcome rather than the overall clinical reasoning process) [19–22]. To the best of our knowledge, no unified nor overall understanding of clinical reasoning exists within the context of the EMS. Thus, the aim of this integrative review is to investigate the clinical reasoning of EMS clinicians and the factors influencing said reasoning.

## Method

### Design

An integrative review is a comprehensive methodological approach used to describe a phenomenon, in this case clinical reasoning. It allows for the inclusion of studies using diverse methodologies [23]. In this study, a 5-stage process was implemented, comprising the following stages: problem identification (presented in the Introduction), literature search, data evaluation, data analysis, and the presentation of results [24].

### Literature search

A systematic and comprehensive literature search was conducted using the Preferred Reporting Items for Systematic Reviews and Meta-Analyses (PRISMA) guidelines [25]. This phase was conducted between November 2017 and January 2018 using 6 established publication databases and indexing services: the Cochrane Library, the Cumulative Index of Nursing and Allied Health Literature (CINAHL), PsycINFO, PubMed, Scopus, and the Web of Science. These databases and indexing services were considered sufficiently extensive in terms of the information they provided, as they span several research disciplines, including nursing, medicine, psychology, cognition, and social science. Search terms were obtained from the Medical Subject Headings nomenclature and, with the appropriate database-specific terminology (i.e. CINAHL headings, categories, and keywords), were combined to enhance the breadth and depth of the search. The literature used to help write the Introduction was a source of eligible search terms. The appropriate search terms were discussed by the authors before a general consensus was reached. Search terms and additional wildcards were searched for within the full scope of the databases’ publications. An experienced librarian provided guidance and support during the initial searches. The full search strategy with search terms and number of hits is presented in Table 1.

**Table 1:** Literature search strategy and number of hits in the databases searched

Search terms/ databases	CINAHL	Cochrane Library	PsycINFO	PubMed	Scopus	Web of Science	Total
Ambulance OR EMS OR prehospital emergency care	14,727	6,195	9,061	27,373	53,629	21,881	132,866
Clinical reasoning OR clinical decision- making OR thinking skills OR critical thinking OR judgment OR decision-making	64,396	10,780	341,925	163,626	4,546	568,985	1,154,258
Total	79,123	16,975	350,986	190,999	58,175	590,866	1,287,124
Combined search terms	CINAHL	Cochrane Library	PsycINFO	PubMed	Scopus	Web of Science	Total
Ambulance OR EMC OR prehospital emergency care AND clinical reasoning OR clinical decision- making OR thinking skills OR critical thinking OR judgment OR decision-making	791	8	462	1,015	30	1,373	3,679
Relevant articles	36	2	16	17	5	62	138

### Inclusion and exclusion criteria

All types of studies were considered eligible, in accordance with the methodology of integrative reviews. The initial inclusion criteria of the studies were: 1) title or abstract describing clinical reasoning in the context of the EMS; 2) published between January 1, 1980 and December 31, 2017; and 3) abstract written in English. This extensive time frame was selected because, to the best of our knowledge, there are no reviews of this specific topic (i.e. clinical reasoning in the context of the EMS). The exclusion criteria comprised records describing air- or water-based EMS, this since the authors expected these to have additional issues influencing clinical reasoning than do road-based EMS.

**Fig. 1:**
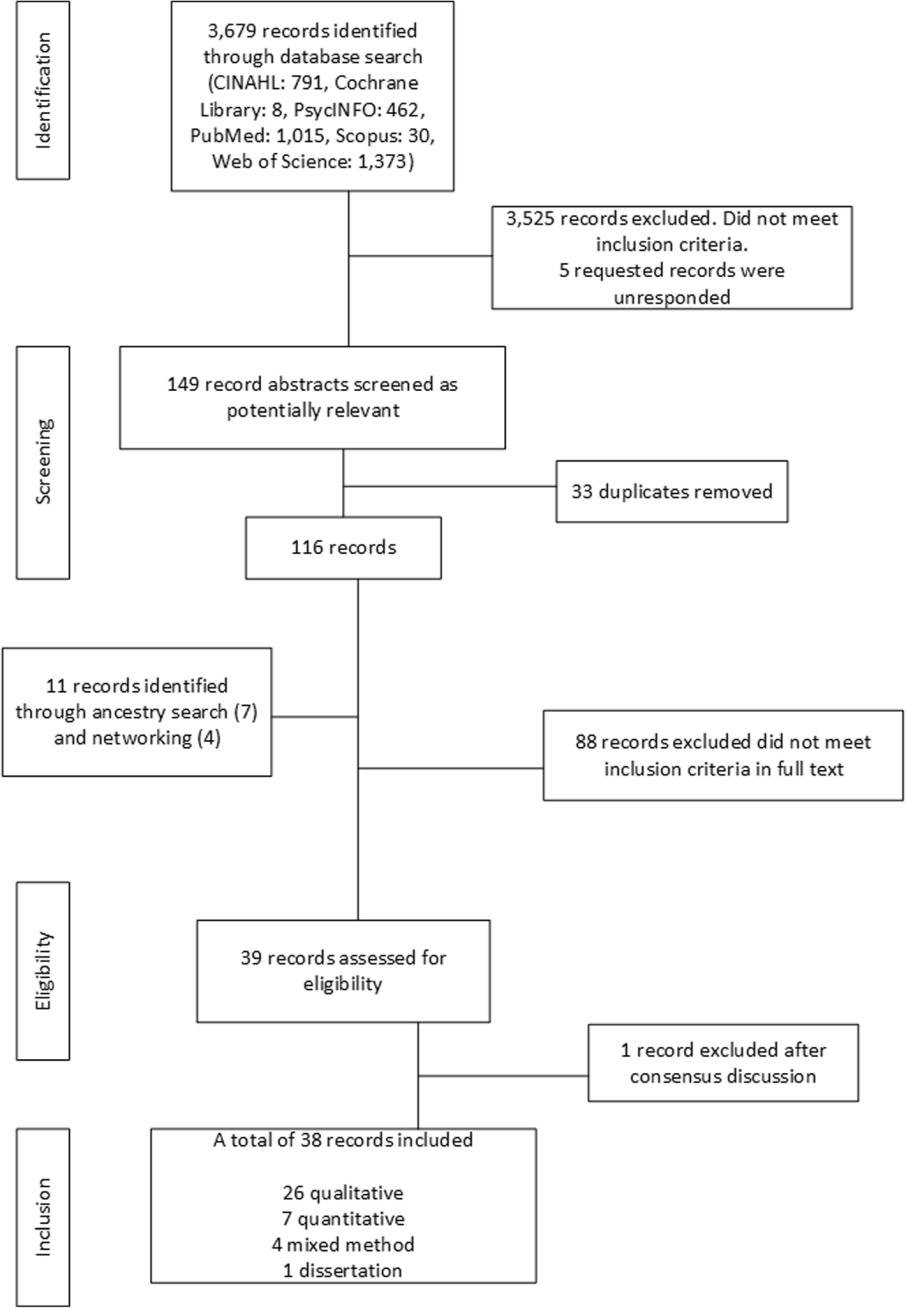
The data evaluation process

The literature search in the electronic databases with combined search terms provided 3,679 records (Table 1) which were screened according to the data evaluation process (Fig. 1). Five of these were unavailable, and requests were sent to the corresponding authors to obtain them, but we received no replies. Initially, 3,525 records did not meet the inclusion criteria, and 33 duplicates were removed. The remaining 116 records then underwent further eligibility screening, in which the full text of each record was read. An additional 11 records were found by personal and professional networking and conducting ancestry searches (i.e., searching through the reference list of records included for additional articles matching our RQs), resulting in 127 records. During the screening process, 88 records were excluded, as the full text of these records did not cover clinical reasoning in the context of the EMS. The remaining 39 eligible records were inserted into an Excel spreadsheet (Table of Evidence – Records Included: Additional file 1) by the first author and then given to the co-authors for additional screening. Following a discussion, 1 record was excluded due to being inadequate in its presentation which made it impossible to interpret the results of the article. Hence, from a total of 3,690 screened records (Fig. 1), 38 were deemed eligible for inclusion and analysis.

### Data evaluation

A critical appraisal tool was used to score the quality of the 38 records included on a four-graded scale [26]. No records were excluded due to a low score in this process; rather, these records contributed, albeit to a lesser extent, to the overall analytic process. The quality scores are presented in the Table of Evidence – Records Included (Additional file 1).

### Data analysis

Data was analysed using an inductive thematic analysis for identifying, analysing and reporting patterns from the data [27]. The analysis process was conducted in six steps. The first step of this analysis was to read and re-read the records included in order to gain an understanding of their content as a whole. In the second step the researchers started generating codes to descriptions which related to the two research questions (i.e. RQ1: the clinical reasoning process and RQ2: the influencing factors) posted for this review. The coding was carried out using a computerized software programme, ATLAS.ti [28]. The coding resulted in 150 codes for RQ1 and 391 codes for RQ2. The third step of the analysis process was subsequently to compare the codes for similarities and differences and to arrange them into main themes. For example, a description of EMS providers experiencing difficulties in using clinical reasoning due to bystanders’ interference was initially categorized under RQ2, and then given the code ‘bystander’.

The fourth step of the analysis process was to review the themes and refine those needing refinement. One example of refinement was changing codes that did not match the theme. Another was restructuring a theme that was too extensive into two smaller themes. However, there are no rules for how many codes may be connected to form a theme and neither is one theme more important than another [27]. After extensive discussions between the researchers a consensus was reached on codes and themes.

The fifth step of the analysis was to define and name the themes; that is, the researchers discussed the actual meaning of the themes and created a narrative explaining the ‘story’ of the theme. The researchers considered the themes in themselves and in relation to other themes. Themes that were too large and complex were broken down into sub-themes to give them structure. For example, the sub-theme coded ‘bystander’ might involve a bystander either aiding or hindering clinical reasoning.

The final step of the analysis process was to create an overarching theme encompassing the coherent meaning of all the codes, sub-themes and main themes. During this step the researchers also wrote up the report. During the analysis, there was constant reassessment of the codes, sub-themes and main themes regarding their coherence and suitability. The themes formed the basis of the results presented in this review.

## Results

The results of the literature search provided 3,690 records. After an eligibility screening, 38 records were included in this review. These rendered a total of 541 codes: 150 relating to how clinical reasoning is conducted (RQ1) and 391 relating to the influencing factors (RQ2). The studies included employed 26 qualitative methods, 7 quantitative methods, and 4 mixed methods. Overall, the results reported in this review built on empirical data from 2,356 EMS clinicians representing 13 different countries.

**Fig. 2:**
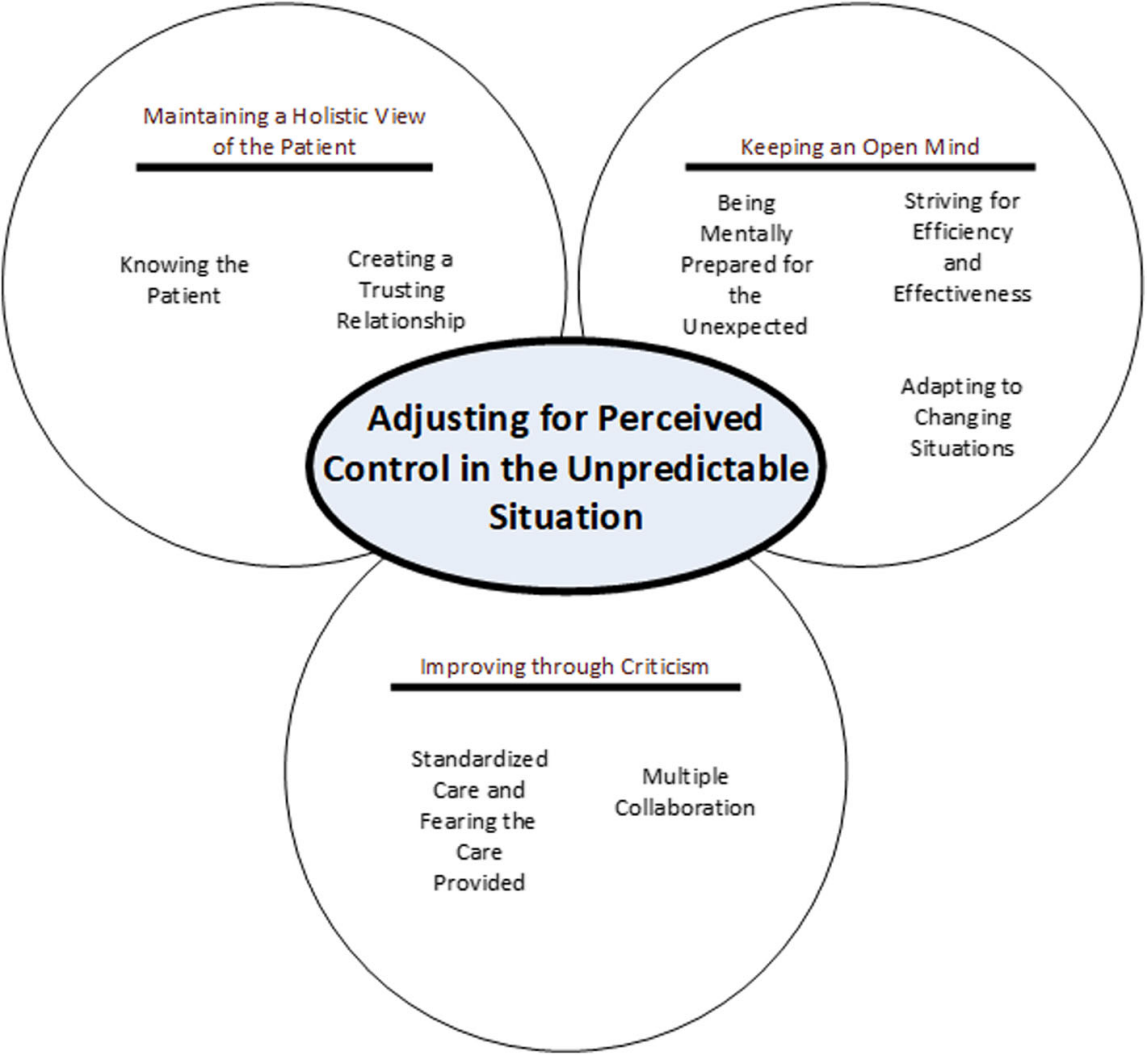
Figure map of the thematic analysis

The analysis resulted in an overarching finding, namely the importance for EMS clinicians to adjust for perceived control in unpredictable situations (Fig. 2). EMS clinicians’ clinical reasoning begins before they even encounter the patient. This beginning is based on the information they receive or do not receive from the dispatch centre. By conducting repeated assessments and planning contingencies for caring, they strive to gain perceived control over the situation in terms of comprehensively understanding the patient and her/his current situation. They need to be constantly aware of what is happening in front of them while foreseeing possible issues that may need to be addressed rapidly and adapting to the issues that arise. This awareness is essential to ensure that they do their work safely and effectively. The awareness is not only important for patients and their relatives, but also for the EMS clinicians themselves, their organizations, and the wider community.

### Three main themes

The results of the analysis were further organized into three main themes: (1) maintaining a holistic view of the patient; (2) keeping an open mind; and (3) improving through criticism. As illustrated in Fig. 2, these themes contain seven associated subthemes. Main themes are written in bold and italic font while their associated sub-themes are written in italic font.

#### Maintaining a holistic view of the patient

This theme illustrates how a holistic view of the patient is created and maintained through understanding both patient and situation and creating a trusting relationship. To make informed decisions regarding patient treatment and the correct course of action, the EMS clinician needs to know what has happened, what the patient and bystanders experience as needed in terms of care, and what signs or clues in the patient’s surrounding environment might tell them. Hence a holistic view is gained by obtaining information from different sources concerning the patient and combining this information with a systematic assessment.

##### Knowing the patient

This sub-theme describes how the EMS clinician strives to obtain a comprehensive picture of what has happened to the patient, why it happened, and the most suitable way to proceed. This is achieved through collecting information, mostly through communicating directly with the patient, a relative, or a bystander [29–37]. The condition of the patient is continuously assessed through dialogue, eye contact, physical touch, and monitoring technology [9]. The patient’s emotional and behavioural responses are assessed as well as those of possible relatives and bystanders [9]. A systematic examination, such as the “head-to-toe,” is carried out and combined with an anamnesis and any information spontaneously provided by the patient, relatives, or bystanders. The information is then verified through more focused examinations [9, 36, 37].

As previously reported [38–40], experienced EMS clinicians carry out more assessments than inexperienced EMS clinicians, especially physical pulmonary assessments and anamnesis. Furthermore, less-experienced EMS clinicians carry out more focused assessments (e.g. focusing on myocardial infarction in a patient with chest pain), while experienced EMS clinicians’ assessments are broader in scope (e.g. considering multiple conditions or diagnoses and narrowing it down bit by bit, rather than focusing on just one diagnosis and working with it until rejected/concluded). Points of interest in the assessment comprise underlying medical causes and/or environmental factors preceding the illness or injury [9, 41]. Information such as this is obtained through anamnesis and an assessment of the patient’s social and environmental circumstances (e.g. home or family situation). In this context, a patient’s surrounding environment is an important aspect of this information, as it may provide clues about everyday life and ability to cope. This is especially important when the patient is not deemed to need hospital care [29, 34, 42, 43]. However, there are also descriptions of occasions on which medical history was not obtained and medical examinations not conducted [37].

##### Creating a trusting relationship

This sub-theme describes EMS clinicians’ ability to create a trusting relationship with the patient and/or relatives. Patients and relatives who trusted the EMS clinicians were more cooperative, shared more accurate information, and respected the EMS clinicians’ opinions [34, 44, 45]. There seems to be a correlation between the patients’ and relatives’ willingness to share information and their educational levels, with higher education or a health-care-related education often being a facilitator of sharing [44].

Personal attributes of both the patient and the EMS clinician influence the process of clinical reasoning. These attributes include age, sex, and physical health status as well as the individual values of the EMS clinician. This can be exemplified by a clinician who attended a course on assessing myocardial infarction and was more proficient in conducting assessments as a result of taking this course. Another example concerns EMS clinicians who were physically influenced by fatigue, which resulted in poor decision-making regarding assessment and treatment [30, 35, 43, 44, 46–51]. The socioeconomic status of the patient was described as not directly influencing the treatment provided. However, socioeconomic status did reflect personal values [35, 44, 47]. EMS clinicians often acted in accordance with how they would have liked to have been treated [49], but they also strove to gain an understanding of the patient’s wishes in order to act in the patient’s best interest [9, 31, 33, 51, 52]. Clinicians need to be able to understand patients’ and relatives’ perspectives, cultural beliefs, and any perceived or expressed wishes regarding medical treatment or the withholding of it. However, it was noted that the more critical a patient’s health condition was, the less the patient’s own views were considered [9, 34, 43, 44, 48, 49]. While EMS clinicians actively seek to involve patients and/or their relatives in decision-making [31, 52], it is also important that they pay attention to their relationship with the patient and any conflict or tension arising [9].

Patients’ relatives are often involved in the decision-making process, sometimes even more than the patients themselves. Relatives may be of assistance but can also hinder the EMS clinicians’ work and create cause for concern. In a desperate situation, they might ask or demand that EMS personnel convey the patient to hospital or begin treatment on site [9, 30, 31, 35, 43, 44, 48–51, 53–56]. Close identification with patients or their relatives might cause EMS clinicians to lose their objectivity, thus increasing the risk of prolonging treatment efforts [29, 34, 53]. In addition to this, the perceived expectations of relatives or other bystanders, such as witnesses, dispatch operators, firemen, or physicians, may influence EMS clinicians’ decision-making. It was noted that public areas caused EMS clinicians to be mindful of their actions and how they communicated, and they often felt compelled to convey the patient to hospital or administer drugs since they thought this was expected of them [30, 48–50, 56, 57].

#### Keeping an open mind

The second main theme describes the ability of EMS clinicians to keep an open mind and to work, provide care, and ensure safety and success in unpredictable situations (e.g. a sudden change in the patient’s condition or a threat arising to clinician or scene safety). This means that they must be prepared for what they are about to encounter, while also keeping an open mind and being able to adapt to any sudden changes. EMS clinicians seem to handle unpredictable situations by creating several plausible scenarios based on limited information.

##### Being mentally prepared for the unexpected

This sub-theme describes how there seems to be a general description in the data of the work done by EMS clinicians. This general description states that EMS clinicians only work with patients suffering from life-threatening conditions. While this seems to be the common view in the medical community and is further reinforced by the organizational culture of the EMS, it is not in agreement with the reality of how the EMS work in practice [41, 43]. Higher levels of education seem to provide EMS clinicians with a more realistic view of what EMS work consists of. They also legitimate the provision of care to patients not in urgent need of treatment in terms of these patients also being important and a common part of EMS work. However, it appears that offers for additional training and education within organizations are optional and mostly rely on individuals investing their personal time [32, 40, 41, 57]. Experience and knowledge are thus not limited to the workplace context: they expand to EMS clinicians’ everyday lives [39].

Keeping an open and reflective mind is described as being extremely important in order to be mentally prepared for the unexpected. EMS clinicians try not to be governed by predetermined statements or information from the dispatch centre. While the information obtained from dispatch centres may put clinicians on the right track, thus giving them the opportunity to prepare mentally for what they are likely to encounter, it can also be inaccurate. This means that clinicians may encounter a completely different and unexpected scenario. Even lack of information is open to interpretation [29, 30, 32, 33, 40, 45, 52]. Appropriate mental preparation often includes determining the receiving hospital and the equipment to bring from the ambulance [33, 58]. First impressions when encountering the patient are essential in assessing whether or not the patient has a serious health condition. The first impression also dictates which assessments to carry out and in which order. A patient who needs urgent treatment facilitates decisions concerning immediate transportation, while a patient who does not need urgent treatment may cause EMS clinicians to reduce their work pace and be more analytical in their clinical reasoning. This may lead an EMS clinician to decide not to convey a patient to hospital [32, 33, 43, 44, 49, 55, 58].

Information is the key element of good decision-making. However, the information must be accurate and presented at the right time. Too little information may lead to the selection of a care pathway that is not appropriate for the patient’s needs. Too much information may cause information overload, leading to confusion [40, 42, 50]. There also seems to be a problem with information generated by EMS clinicians themselves (e.g. a symptom that has not been presented by the patient but is still treated and reported as true). There were also reports of assessment results, even though no actual assessment had been conducted [29, 36, 37]. These cases seem to be more common in highly stressful situations.

The creation, verification or rejection of hypotheses seems to be the method used by most EMS clinicians. This is done through a systematic procedure of anamnesis and parallel examinations. During this procedure, additional hypotheses may be produced, based on new findings and assessments. In addition, EMS clinicians sometimes do a mental simulation of the options considered to weigh and evaluate all possible consequences [32, 36–39, 55, 58–60]. The number of hypotheses depends on the situation and on the experience of the EMS clinicians in assessing patients. More experience provided a higher specificity in patient hypotheses [32, 37–39, 42, 55]. A lack of experience of specific diagnoses sometimes resulted in a feeling among clinicians of being ready to act but not being confident in the situation [32].

##### Striving for efficiency and effectiveness

This subtheme describes how the safety of both EMS clinicians and their patients is of primary concern when striving for efficiency and effectiveness. This safety zone seems to be more important in public settings than in a patient’s home [32, 45, 49, 50, 61]. Creating a safety zone means that environmental factors are assessed for potential threats to safety. This assessment often begins by assessing any potential uncertainties in terms of the location of the patient and the ongoing process of assessing and evaluating her/his safety by scanning both surroundings and bystanders [9, 58, 61]. When EMS clinicians are in confined and unfamiliar environments, potential or actual threats may intensify, especially when dealing with patients who are unpredictable [9, 35, 49]. Patients’ physical attributes, such as extreme obesity, may also represent a potential safety risk, as lifting them could lead to EMS clinicians becoming injured and result in their extrication from the scene [30, 56].

More experienced EMS clinicians claim that being a mentor for those with less experience or education is a way of creating a safer work environment. Some of the tasks addressed by mentors included situations where colleagues communicated inappropriately, omitted or did not carry out procedures or examinations thoroughly, handled equipment in an incorrect manner, underestimated the seriousness of the patient’s condition, and did not ask for assistance [32, 58]. Guidelines and decision-support tools may be of assistance in chaotic and time-sensitive situations, since these tools and guidelines are not influenced by bias (e.g. the emotional state of the user). They also reduce the inappropriate use of mental shortcuts [54, 60].

##### Adapting to changing situations

This subtheme describes the ability of EMS clinicians to adapt to the surrounding environment on the scene - patient, relatives, and bystanders. This adaptation appears to be crucial for clinicians and may consist of interacting socially (e.g. with colleagues, other personnel at the scene, the patient and relatives), solving problems presented by physical elements (e.g. how to transport the patient from a car wreck to the ambulance) and assessing human elements (e.g. assessing bystanders’ abilities to assist or hinder their work). An open and reflective mind-set makes adapting to sudden changes possible and allows EMS clinicians to shift their focus between different points of interest [32, 39, 45, 61, 62]. This mind-set makes it possible for them to consider multiple solutions to a clinical problem. As a result, they may not be completely governed by established rules and guidelines but instead be amenable to a more situational response [32, 39, 45, 53, 60]. Uncertainties arise from different factors present in the situation rather than from the situation itself [49, 58]. This can be seen in terms of the age of a patient being a factor in the decision-making process, with greater effort being made in the treatment of children and younger patients [30, 35, 47, 49, 50].

Education and regular training in combination with accumulated experience enable EMS clinicians to internalize their knowledge. Being able to make use of this knowledge is of great importance, as they often work in relative isolation with limited collegial support. It was suggested that education was a major factor contributing to EMS clinicians’ sense of confidence in assessment and decision-making. Those with higher education are more prone to look for and use additional sources of information; higher education also enhances their reasoning skills and thus, they can make reasonable decisions more easily [29, 30, 32, 34, 35, 39–42, 44, 49]. Furthermore, experience helps them to deal with stress and allows them to process different facets of information simultaneously. Experience also provides clinicians with the confidence to realize that things do not always go to plan, and it gives them the courage to admit when they are wrong [30, 32, 39, 58]. EMS clinicians use their own judgement in combination with decision-support systems provided by their organizations. Depending on their experience, they do not always carry out all of the stated steps within a guideline but rather use chosen aspects of it [9, 30, 32, 37, 39, 40, 52–54, 60]. This relates to the view that clinical reasoning and decision-making are not linear processes and that guidelines are often too strict and thus not easily applicable to a given EMS context [9, 32, 33, 41, 49, 60].

#### Improving through criticism

This main theme describes how the skills and knowledge base of EMS clinicians are improved through education and collaboration, and also how they relate to the standardized care guidelines of their organization. These guidelines may help them or be a cause for concern.

##### Standardized care and fearing the care provided

This sub-theme describes how EMS clinicians relate to the standardized care protocols that govern their work. Clinical practice guidelines and electronic decision tools are the main decision support systems available. The latter provide an understanding of what is expected in a certain situation or condition. The former provide the steps involved in recommended assessments and guide the decision-making process [30, 32–34, 54, 60]. However, these systems are sometimes viewed as only being needed by beginner clinicians or for educational purposes. The guidelines may also create obstacles (e.g. when the guidelines state that EMS clinicians should begin resuscitation even though they believe it to be pointless) [9, 32, 33, 37, 41, 49, 52, 53, 60]. These flaws and obstacles mean that EMS clinicians must rely on their own judgement more often and thus potentially expose themselves to criticism [32, 41, 52–54].

EMS clinicians work under the fear of criticism (e.g. if they do not follow protocol, it could lead to disciplinary measures). This fear means that EMS clinicians often convey patients to hospitals, even though certain patients could well have stayed at home or been treated in a more suitable environment. Most organizations seem to have limited support systems for individual EMS clinicians in these situations, and there is a “blame and shame” culture within EMS organizations. The lack of support and the aforementioned culture act as barriers to incident-reporting rather than presenting an opportunity to learn from adverse events [29–31, 40, 41, 44, 54, 57]. Socioeconomic status was another factor that influenced the clinical reasoning process, especially in countries where patients must have financial means to receive care (i.e. clinicians need to address these issues before making decisions about treatment) [44, 47].

##### Multiple collaborations

Teamwork and collaboration is not only achieved by colleagues in the EMS but also with the patient, relatives, or other personnel working at the scene. When in doubt, EMS clinicians seek confirmation from others, discussing their preferences with colleagues or other healthcare professionals in order to find a suitable course of action [9, 30–32, 36, 39, 43, 45, 49, 62]. Through discussion and feedback with colleagues or other professionals, EMS clinicians can improve and develop their knowledge and skills, thus providing their patients with suitable care [9, 30–32, 36, 40, 49, 50, 53]. However, these discussions with other healthcare personnel can often be challenging, due to their general lack of knowledge of EMS clinicians’ skills and responsibilities. This may make referrals and handovers more difficult [32, 40, 43].

Previous experience of teamwork is described as important for improving collaboration, both within EMS teams and with immediate colleagues and other personnel [49]. Working with a colleague from the same specialization means working with an equal, and it also reduces the risk of being limited to a particular mode of thinking or tasks being forgotten. This seems to be important in the context of the EMS, since those with a non-EMS specialization often apply hospital-adapted care which is often inadequate in a prehospital setting. Furthermore, EMS clinicians’ communication (especially non-verbal skills) and cooperation skills improve if they know each other [32, 49]. More experienced EMS clinicians made more use of their colleagues by delegating tasks [38].

## Discussion

This review shows that EMS clinicians adjust themselves and their strategies for clinical reasoning in order to gain a perceived control in an unpredictable situation. Their clinical reasoning is influenced by a great number of factors where correct information, communication and trust are key for planning and executing good treatment and care. Clinical reasoning seems hard to capture, especially EMS clinicians’ thoughts and emotions and how these influence the clinicians.

The group of authors are experts in various disciplines, including prehospital emergency care, emergency nursing, anaesthesia care nursing, caring sciences and information sciences. The authors’ experiences of the research field in terms of clinical and theoretical knowledge can be considered both a strength and limitation. The limitation considered here was managed through a number of open discussion throughout the review process in order to raise awareness of possible ‘blindness’, flawed thinking, or bias during the research process. Another limitation, despite its also being a main motivator for the study, was the lack of general descriptions of clinical reasoning in the context of the EMS. This lack made it difficult to find literature on the subject, since there was no common terminology. A third limitation in relation to this review was the difficulty of selecting search terms. Literature searches in electronic databases is said to only generate about 50% of all potentially eligible literature [24]. However, this study may be considered fairly conclusive since it is based on a large data set found through a number of well-established electronic databases. The data set mostly represents a higher-income Western countries. Representation from countries with other values, cultures or low income levels might have reflected other issues influencing the clinical reasoning of EMS clinicians.

From the results of the review, three discussion themes emerged: (1) Clinical reasoning and the situation with biases; (2) the use of workarounds to help EMS clinicians solve various issues; (3) the lack of organizational support which creates fear of blame and shame for any mistakes made.

When discussing clinical reasoning, Croskerry [63] argues that comprehensive medical knowledge is needed to make good decisions regarding a diagnosis, but knowledge alone is not sufficient for clinical reasoning. EMS clinicians need to know *what* to think, but they also need to understand *how* to think and to understand how they feel, reason, solve problems, and make decisions. From the results of this review regarding how clinical reasoning is conducted, it is clear that EMS clinicians use different methods of thinking to solve certain issues. The first method is an unreflective and rapid response (e.g. when quickly adapting to a changing situation or when working under stress). It is a quick process that is mostly used in life-threatening or routine situations. The second method of thinking involves a slower but more reflective and analytic process, which is used in complex or non-routine situations (e.g. when making structured assessments to develop a theory of plausible diagnosis). When studied within other disciplines, organizations, and contexts [64], the context in which decision-making takes place was found to have great influence these methods [65]. Hence, there are many factors that contribute to the decision-making process and influence its outcome [29, 64–68]. Unfortunately, however, most of the research on clinical decision-making processes was conducted outside the context of the EMS and was instead conducted in controlled environments (e.g. simulations) [69–71]. This type of environment is preferred when examining different factors on an individual basis, but in the context of the EMS, there is a complex interplay between many factors that continuously contribute to the work and care process.

Cognitive biases are described as dispositions that influences the way a person thinks [72, 73], and there are several biases directly mentioned or described in other words in the data of this review. Awareness and recognition of cognitive biases are seen as hallmarks of rationality; not recognizing bias creates irrational behaviour, leading to these biased issues remaining unaddressed [74]. The need exists to address these issues in all healthcare organizations, particularly in the context of the EMS.

EMS clinicians often seem to be creative when striving to solve issues arising during their work (e.g. when guidelines cannot be used as stated). As reported in the results, guidelines may offer limited guidance on how to solve these issues, hence the need for alternative strategies or approaches, such as workarounds. Workarounds are described as “observed or described behaviours that may differ from organisationally prescribed or intended procedures. They circumvent or temporarily ‘fix’ an evident or perceived workflow hindrance in order to meet a goal or to achieve it more readily” [70]. Regardless of the factors that contribute to workarounds, their use bypasses system processes that are in place to assure patient safety [71]. Workarounds are used both individually and collectively and are most often viewed negatively. However, while they are deemed necessary to implement high-quality care successfully in some contexts, they are counterproductive in others [70]. Since EMS clinicians often lack contextualized guidelines and provide care in a constantly changing environments, the EMS may present a context in which workarounds may be viewed as a necessity.

The results indicate that EMS clinicians strive to provide care for their patients to the best of their ability, often with the patients’ preferences accounted for. Demands, sometimes conflicting, are made on individual EMS clinicians from many sources, including themselves, patients and their relatives, EMS organizations, and society as a whole. This leads to a feeling of isolation in terms of making assessments and decisions in order to remedy a situation. EMS clinicians carry out these assessments and make decisions without sufficient resources or supportive tools, particularly organizational support. It appears that EMS clinicians comply with guidelines provided so as to avoid disciplinary measures. This is also the case when EMS clinicians feel uncertain of how to proceed. Thus a blame and shame culture exists in the EMS [75] and other healthcare organizations [76]. Awareness of this blame and shame culture and its negative effects provide a basis for addressing the issue. It was previously suggested [75, 76] that those who manage EMS organizations should examine the problem from a system-based perspective, examine the factors that have led to adverse events, and make changes in the system so that the same events do not happen again. However, even though a system-based approach is forgiving, it still means that an individual EMS clinician is held accountable for grossly negligent actions.

This review adds to greater and more summarized knowledge base regarding EMS clinicians’ clinical reasoning and what influences it. This since the review presents a collective view and description of the major parts of the clinical reasoning process and factors influencing it, a great number of factors were the same even though the location, patient or EMS team differed. However, more research is needed and in order to draw any real conclusions it needs to reflect the work processes of local EMS organizations. There are many questions that still need answers, but based on this review, further research should aim at improving EMS clinicians’ clinical reasoning and reducing the negative influences of biases. Research done out in the field is essential, since it is only where the actual clinical reasoning takes place that we can get close enough to start understanding its function in real life.

## Conclusion

Many factors influence the clinical reasoning of EMS clinicians. It is difficult to address them all at once, but perhaps it is best to begin with the factors that can be addressed with ease as well as those that seem to have a greater influence on the clinical reasoning process and patient outcomes. Making EMS clinicians more attentive to these questions facilitates open discussions with colleagues and superiors. Further research is needed into the factors that influence the clinical reasoning of the EMS in the field. Additional research should aim to investigate which factors are the most influential through observations or by interviewing EMS clinicians.

Finally, the following steps need to be taken:

- awareness of cognitive biases and their influence on clinical reasoning must be raised;
- the blaming and shaming culture in EMS organizations must be inhibited; and
- learning opportunities from adverse events must be created and shared with other EMS organizations.

## Additional file

Additional file 1: Table of Evidence - Records Included. (DOCX 115 kb)

## Data Availability

All data generated or analyzed during this study is included in this published article (and its supplementary information files: Additional file 1 – Table of Evidence – Records Included). Also, the datasets used and/or analyzed during the current study are available from the corresponding author on reasonable request.
