# Supplementary material for: Clinical reasoning in the emergency medical services: an integrative review
Source: Scand J Trauma Resusc Emerg Med. 2019 Aug 19;27:76. doi: 10.1186/s13049-019-0646-y (PMC6700770; doi:10.1186/s13049-019-0646-y)
Supplement: Supplementary file 1 — Table of Evidence - Records Included. (DOCX 115 kb) [file 13049_2019_646_MOESM1_ESM.docx]

| Article | Title | Citation | Methods | Aim | Results | Limitations | Quality of Evidence (Good, Fair, Poor, Very Poor) |
| --- | --- | --- | --- | --- | --- | --- | --- |
| 1 | A qualitative study of systemic influences on paramedic decision-making: care transitions and patient safety | O’Hara, R., Johnson, M., Siriwardena, A et.al. *Journal of Health Services Research & Policy, 2015; 20(suppl.1): 45-53* | Qualitative multi-method.  Interview,  Non-participant observation  Digital diaries (audio)  Focus groups  Documents (annual reports, policies and protocols)  3 Ambulance Service Trust in England (NHS), paramedic and specialist paramedics (n=50). | **Aim:** To explore system-wide influences on decision-making by paramedics, focused on care transitions and potential risk factors. | **Results:** revealed the complexity of transition decisions and system influences potentially impacting on patient safety.   - Transitions decisions range from clear-cut emergencies, including protocol-driven decision for e.g. STEMI or trauma to more complex cases where the patients’ social circumstances and co-morbidities need consideration. The latter type created most uncertainty and risk for both patient and paramedic. - Shift in the demand for ambulance service care has impacted on the scope of clinical decision-making as the profile of calls shifted from primarily emergency care decisions to now dealing with a wider range of primary care and psychological decisions. Non-conveyance decisions are more time-consuming and required a high level of skill and support. - The increase in non-emergency was perceived to dilute exposure to life-threatening emergencies which might contribute to skill degradation and risk regarding time-critical emergencies. - Organizational pressure of meeting performance indicators constituted a stressor for paramedics. Non-conveyance decisions are more time-consuming than conveyance. - Decision-making regarding appropriate care options may be frustrating when paramedics lack access to them. Especially when out-of-hours, weekends and bank holidays. Not all areas have alternative care pathways, they may fluctuate across the Trusts. - Non-conveyance was considered a risk for both patient and paramedic. Risk tolerance was influenced by competence, confidence or negative experiences. Conveyance could be seen as a safety net for paramedics. - Paramedics had a low confidence in organizational support in the event of an incident. “Blame and Shame”, rather than learning led to a barrier for incident-reporting. Cultural barrier for improving service delivery and patient safety. - Paramedics wanted additional training to benefit their competence and confidence supporting their decision and communication. Most offers for training were optional, relying on personal investment of time. - Most paramedics though that other healthcare professionals were unaware of their enhanced skills and responsibilities making communication and referrals difficult. - Decisions are based on partial knowledge of potential options when decision support is limited. - Paramedics use a range of passive support systems such as pathway algorithms, decision aids for assessments. Some had an electronic access for local services clinical guidelines. - Experience and thoughts about more active support systems like “clinical hubs” were mixed. - Dispatch information had the potential to inform and frame the crew, although limited and potentially misleading. - Keeping an open mind attending “frequent” callers. - Information overload. - Lack of constructive feedback limited opportunities to reflect and learn. - Variations in access to equipment and drugs have the potential to impact on decisions about patient care. - Tension between service demands and availability of resources were a source of pressure for staff. | **Limitations:**  A relatively small-scale qualitative study. Not including any patient safety measures. Self-selected sample of paramedics (n=50).  **Strengths:**  Multiple methods provide consistent evidence around key issues. Data was compared from both insider and outsider perspectives. | Fair |
| 2 | A survey to determine decision-making styles of working paramedics and student paramedics | Jensen, J., Bienkowski, A., Travers, A., et.al. *Canadian Journal of Emergency Medicine(2016);18(3):213-222* | Cross-sectional paper-based survey.  Participants: working ground ambulance paramedics and student paramedics.  The REI-40-instrument is a psychometric toll for identifying thinking styles. | **Aim:** To determine paramedic and paramedic student preferences toward, and perceived ability to use, experiential and rational thinking styles, and to determine whether thinking styles differ between these two groups. | **Results:** 1172 surveys were included (904 WP, 268 SP)   - Paramedics scored high on rational thinking style over experiential. - Paramedics who were males, younger, fewer working years, experience, over a year since last training opportunity, and who worked in urban or mix of urban-rural settings scored higher on rational questions than their counterparts. - No differences between participants regarding rational thinking were found in type of paramedic training completed or hours worked per week. - No differences were found in the participants concerning scores on experiential thinking. - Paramedic students also scored high on rational thinking. - Rational thinking styles were given higher scores by younger who had more prior education compared to their counterparts. - No difference was found among the students in experiential thinking scores. - No difference between males and females in rational thinking. - Females scored higher on experiential thinking than males. - Younger scored higher on rational thinking than older, however there was no difference in experiential thinking scores. - Both participant groups scored above midpoint on experiential thinking styles, which may indicate that they use both thinking styles. (Dual process theory). - Rational thinking scores were higher among the student group than the working group.   **Conclusion:** Both working and student paramedics report that they prefer and perceive that they have the ability to use rational over experiential thinking. | **Limitations:** The REI-40 instrument captures self-reported perception of ability to use, preference toward, rational and experiential thinking and may not be an accurate representation of thinking styles in an actual clinical setting.  No meaningful important differences between scores has been established for the REI-40 which makes it hard to draw conclusions between differences in score and thinking. | Good |
| 3 | Balancing between closeness and distance: emergency medical services personnel’s experiences of caring for families at out-of-hospital cardiac arrest and sudden death | Bremer, A., Dahlberg, K., & Sandman L. *Prehospital and Disaster Medicine, 2012;27(1):42-52* | Qualitative interviews with hermeneutic lifeworld perspective.  10 EMS personnel. | **Aim:** To analyze EMS personnel’s experiences of caring for families when patients suffer cardiac arrest and sudden death. | **Results:**   - Ethical dilemma when switching from/between life-saving to caring for family members. - Lack of official decision-makers or enforcers. - The moral responsibilities are perceived as high in cardiac arrest and sudden death incidents. - Formal guidelines might direct the EMS to the next mission before they could help the family members to closure. But there is no formal responsibility, if any at all, for when or to which extent EMS personnel should care for family members. - Responsibilities seems to be shaped by circumstances like context of judgements, perceptions, feelings and reactions. But are mostly “natural”, instinctive and unreflecting. - In this context the EMS personnel move from well-structured guidance (CPR) to a situational response in which they balance between interpretive reasoning and direct emotional, affective response at their own discretion. - EMS tries to access the family’s state of mind, knowledge of the situation. - EMS personnel’s decisions and actions were influenced by the family’s state of mind. - Decisions regarding terminating CPR may trigger feelings of guilt, failure and betrayal due to failure to save a life. - Close identification with the family’s situation may cause distance to the patient’s objective status and continuation of futile CPR. - Existential escape is a result of EMS personnel’s inability to distance themselves from identification with the family. - Lack of experience seemed to increase feelings of inadequacy, reducing the security of decisions to refrain from CPR. - Feedback and being able to discuss and reflect with colleagues was important. Sharing experiences. - Ethical caring competencies place great demand on flexibility in the decision-making process.   **Conclusion:** EMS personnel felt inadequate when responsibility was extended to caring for family members. Flexibility in the decision-making process is vital. There is also a need for moral education and clinical ethics training. | **Limitations:**  Limited to social, religious and cultural norms of Northern Europe society.  Result might be biased by EMS personnel who perceived care for bereaved families as important.  Limited number of participants.  The interviewers’ pre-understanding of the phenomenon might influence the objectivity. | Good |
| 4 | Being prepared for the unprepared: A phenomenology field study of Swedish prehospital care | Wireklint Sundström, B., & Dahlberg, K. *Journal of Emergency Nursing, 2012;38(6):571-577* | 11 informants, specialist ambulance nurses, registered nurses and paramedics.  Observation with reflective lifeworld approach. Field notes and unstructured interviews. | **Aim:** To highlight prehospital care and how ambulance personnel prepare for their everyday caring assignments and avoid making premature decisions. | **Results:**   - Preparations start with the pre-information from the dispatch centre. But this might be inaccurate and the personnel may face a completely different scenario upon arrival at the scene. - EMS personnel tries to not be governed by predetermined statements by keeping an open mind. - EMS personnel has a desire for control and effectiveness in a practice full of changing situations and surprises. - It is important to be calm and cope with stress in chaotic and time-pressed situations, as well as support patients and the person close to them. - Care in the prehospital environment requires the EMS personnel to be alert, aware and prepared for an open and flexible encounter with the patient, while maintaining their certainty and control. - Assignments with little or no information are perceived as hard. - EMS personnel are dependent on the cooperation of the patient, and the patient’s participation depends on trust for the EMS. - Being able to work without a pre-formulated action plan. - Acting on what is presented in form of medical needs as well as for what might happen during the assignment. Even though EMS personnel sometimes do not know what will happen next, they need to create a calm and positive atmosphere. - A lifeworld-led interaction between patients and EMS-personnel strengthens the medical assessment.   **Conclusion:**   - EMS-personnel need to stay alert and keep an open mind during assignments. This in order to be able to react to sudden changes and adapt. | **Limitations:**  The experiences cannot be applied generally to all EMS-personnel.  Participants were all from the same ambulance station.  Data collection was carried out 10 years previously, although analyses were carried out within 6 months of data collection. | Good |
| 5 | Between professional values and the social valuation of patients: The fluctuating economy of pre-hospital emergency work | Nurok, M., & Henckes, N. *Social Science & Medicine, 2009;68:504-510* | Fieldwork in Pre-hospital emergency services in Paris and New York.  Observations were recorded in a small notebook. These notes were then analyzed using principles of Grounded theory, notes were coded in order to identify basic social processes. Codes were regrouped into categories. | **Aim:** A bit unclear but seeking to resolve different views on how social values influence medical decisions in the EMS in France and USA. | **Results:**   - In both France and USA, age of the patient affected the way in which the case was managed. - Age affects how professionals engage in therapeutic decisions, young patients are more aggressively treated whereas elderly patients or those in poor health often receive less heroic intervention. This was found to greater extent in Paris where professionals are free to tailor treatment according to their clinical judgement. However, in New York the researchers could see that paramedics who are obliged to resuscitate aggressively regardless of age, efforts were often slower and less vigorous for the elderly. - Socio-economic status also influences decisions made during emergencies. Patients judged as low social value got little interest from the paramedics in the USA until they had a confirmed serious condition which they could treat (high value), like an overdose. When treated the patient lost in value again because of the origin of the problem was solved. - Low social value does not necessarily mean that an individual case will be less valuable per se, but social value competes with other values. - Cases with more technical tasks (trauma) were perceived as of higher value to the paramedics in both Paris and New York. - Giving medical advice or simply transporting patients to the ED were considered as low value. - Medical cases (non-trauma) needed more work experience and were considered “harder” than surgical cases. - Saving someone in public is of higher value than out of public, this irrespective of complexity of the case. But most commonly the paramedics remember the ones they saved. - Heroic value is linked to social utility, recognition and self-esteem. - Less experienced paramedics were “hungry” for cases whereas those with experience were not. This might be to improve their skills. - Both attributes of the patient and the case itself can affect the value of it. And this varies between professionals. - The direction of work might be influenced by the gender of the paramedic. | **Limitations:**  The findings are not generally applicable to every pre-hospital or medical context. | Poor |
| 6 | Complexity of the decision-making process of ambulance staff for assessment and referral of older people who have fallen: a qualitative study. | Halter, M., Vernon, S., Snooks, H., Porter, A., Close, J., Moore, F., & Porsz, S. *Emergency Medicine Journal, 2011:28;44-50.* | **Methods**:  Semi-structured interview, asking 11 EMTs and 1 Paramedic in London Ambulance Service NHS Trust to describe their process of decision-making.  Thematic analysis. | **Aim:**  What are the processes used by ambulance staff in the assessment of older people who have fallen?  This to gain greater understanding of these practices following an implementation of a “clinical assessment tool”, in the context of low usage rates identified during the study. | **Results:**  Analysis of the interviews revealed a similar approach to assessment in this particular patient group. It has been split into four stages: 1-prearrival, 2-initial contact, 3-continuing assessment and 4-making a conveyance decision.   - *Pre-arrival*: The assessment and decision-making process was described as beginning with information received from the ambulance call centre, including the labelling of patient’s need as ‘assist only’. This formed a picture of a person who had not sustained any injury even though the majority recognized that the person might have experienced an injury or be medically unwell. The meaning of the word ‘fall’ differed between the participants. - *Initial contact*: Making an assessment of the patient regarding their initial condition (level of consciousness, pain, injuries associated with the fall, how long had the patient been on the floor or patient’s medical condition) the reason for falling was important. Creating a rapport with the patient for trust. - *Continuing assessment*: If there was not any immediate medical need to convey the patient to ED all participants described a further assessment used for making conveyance decisions. This assessment comprised: patient’s medical history, medications, current condition and mobility, and the social and environmental circumstances. Information was gathered from the patient, professional and non-professional carers, and from records in the patient’s home. Information might be affected by the patient’s anxiety regarding being conveyed to the ED, their poor health or cognitive impairment. Vital signs were an important measurement for all participants as was the patient’s mobility. Some participants suggested a reluctance to carry in heavy equipment if access to the home was not straightforward. Assessing the patient’s living conditions with environmental and social circumstances was described as important when making a conveyance decision. Did the patient have someone caring for them, when was their next visit, was there food at home, heating etc.? - *Making a conveyance decision*: The CAT had been completed retrospectively after a decision of conveyance was made. There was an ambivalent attitude to the standardized assessment tool among the participants. The participants described their previous work experience being most influential in their decision-making. Some participants always tried to convey the patient to the ED due to concern about what would happen next to the patient, especially in more complex situations regarding health and social care situations. The participants also described feeling sympathy for the patient and respect for their right to decide whether or not to go to the ED. Participants described the need to ‘cover their backs’ in case of repercussions arising from non-conveyance.   **Conclusion:**  Highlights complexity of decision-making. Most decision-making seems to be informal processes. There is a reluctant to use a systematic decision-making tool. Further research is needed to look at how new care pathways offering alternatives to the ED may influence decision-making around non-conveyance and thus patient health outcome. | **Limitations:**  Small study using a convenience sampling. More EMTs than paramedics. Participants with fewer years’ work experience were slightly over-represented.  All participants had the same formal training in using the assessment tool. | Good-Fair |
| 7 | Decision-making by ambulance clinicians in London when managing patients with epilepsy: a qualitative study. | Burrell, L., Noble, A., & Ridsdale, L. *Emergency Medicine Journal, 2013;30:236-240* | **Methods:**  In-depth interviews with 15 ambulance clinicians working in South London.  Convenience sampling. Thematic coding.  1 EMT level 2, 4 EMT level 3, 5 paramedics, 4 paramedic team leaders and 1 emergency care practitioner. | **Aim:**  To explore the ambulance clinicians’ perceptions of which factors influence their decisions when caring for patients with epilepsy. – How confident are they in managing these patients and what factors influence their decisions on how to care for them and whether to transport them to the ED and not. | **Results:**   - 1/3 of participants reported low levels of confidence when caring for patients with epilepsy. Those who felt confident attributed this to the length of their experience, not their training. Those with less experience felt less confidence and found seizures disturbing to witness. - When asked to describe cases that went well, clinicians typically described emergency states such as status epilepticus. They felt that they were able to carry out interventions in accordance with their training and guidelines were available. They had also observed benefits to the patients, such as termination of seizures. - Situations where the patient had finished a seizure spontaneously or had complex partial seizures were considered more challenging. - 1/3 of participants viewed their training as inadequate to prepare them for such situations. - Even though 2/3 stated that they felt confident in managing patients with epilepsy, only just over half of the participants felt confident enough to assess these patients’ need for transportation to the A&E. Reasons for lack of confidence were feelings of not being able to assess the patient adequately, negative experiences. However, those who felt confident in deciding not to transport were often guided by the views of the patient. - Lack of access to patient history was recognized as a significant challenge for safe triage and transportation. - Fears of litigation were another reason for transporting patients with epilepsy to the A&E as reported by 1/3 of participants. Only 1/3 felt they were assured adequate organizational support in clinical decisions they made for patients with epilepsy. Transportation to A&E was seen as a safety-net for the clinicians. - If the call took place in public there was a feeling of pressure and bystanders’ expectations of transportation and drug administration.   **Conclusion:**  Patients with epilepsy might be transported to the A&E not because of clinical need but because the ambulance clinician often felt insufficiently confident or informed to assess the medical need adequately. This was combined with a lack of alternatives to A&E for continued care.  Further research is needed. | **Limitations:**  A wide variety of skill levels and years of experience is a strength since these factors are expected to influence practice.  The convenience sample is however a limitation in regard to potential bias.  Strengths and limitations may be that participants are reluctant to discuss areas of their practice outside recognized normal protocols. However, they may have talked more openly about their opinions due to interaction with an interviewer who was also perceived as a colleague with shared experiences. | Good |
| 8 | Emergency medical service provider decision-making in out of hospital cardiac arrest: an exploratory study | Brandling, J., Kirby, K., Black, S., Voss, S., & Benger, J. *BMC Emergency Medicine, 2017;17(24):1-8* | **Methods:**  4 focus groups with 16 EMS providers. Discussed four case vignettes to explore decision-making. Thematic analysis was used to analyze transcripts. | **Aim:**  To explore the influences on UK EMS providers’ decision-making when commencing and ceasing resuscitation attempts in OHCA. | **Results:**  EMS provider decision-making in OHCA is influenced by guidelines, protocol and policy.   - The initial call including its information will start the EMS providers’ decision-making. - On arrival at the scene EMS providers will use their clinical reasoning to make a judgement about the feasibility of resuscitation as well as asking for more information from the people present. - Assuming there is a chance of survival or no obvious reasons not to begin resuscitation EMS providers will commence. - EMS providers seem to use factual, seemingly definitive, information to make decisions about resuscitation. Primarily by ECG. But also comorbidities, advanced directives (do not resuscitate), these can be difficult to adhere to when family members are distressed and asking for their loved ones to be saved. - There was considerable discussion between participants in one case where it was believed that a do not resuscitate was in place but could not be found. Some felt worried about criticism or disciplinary procedures if they did not attempt resuscitation where no formal paperwork had been located. - Protocols, guidance and policy provided an understanding of what is expected from the EMS providers. Only one referred to being guided by research when making decisions outside the guidelines. - Distance to hospital was a factor in decision-making, time to transportation, likelihood of survival, viability of on-board resuscitation vs stabilisation in situ, but also needing help to carry out the resuscitation attempt. - The age of the patient had an effect on EMS providers’ behaviour. Children would always be subjects for resuscitation attempts. Age in accordance to comorbidities was also a consideration. - Intrapersonal factors could influence decision-making, respecting other colleagues’ wishes or doubts. - Onlookers, particularity parents were a compelling reason for commencing resuscitation and remove the person from the scene. - Also fear of having to defend decisions made in formal court proceedings was a reason for commencing or terminating resuscitation after consulting a doctor. - Risk factors for the EMS providers influenced the decision to commence resuscitation. E.g., an obese patient who might delay the extrication process but also put the providers at risk of physical injury. - Work experience affected confidence in their own decision-making. And also if the EMS providers felt that their decisions would be supported by their employer. - EMS providers might experience anxiety out of concern for whether they were acting in the interest of the patient or the possibility of litigation and disciplinary actions. - Calls that came at the end of the shift – EMS providers suggested that some would accelerate the decision to extract the person and take them to hospital while others would try even harder at the scene so they would not be accused of doing less at the end of the shift, or doubt their own efforts.   **Conclusion:**  Influential factors that can lead to different decisions and variability in practice are:   - Factual information available to the EMS provider. - Structural factors such as protocol, guidance and research - Cultural beliefs and values - Intrapersonal factors - Risk factors - Personal values and beliefs.   Improved understanding of the circumstantial, individual and interpersonal factors that meditate decision-making in practice can inform the development of more effective clinical guidelines, enhanced education and clinical decision support. | **Limitations:**  Focus group participants may be influenced by other participants’ opinions, in both positive and negative ways.  Even though the cases were based on real-life scenarios, the participants could sit in a calm environment to discuss actions to be made in them. In real-life there would not be so much time for that. | Fair |
| 9 | Consensus on paramedic clinical decisions during high-acuity emergency calls: results of a Canadian Delphi study | Jensen, J., Croskerry, P., & Travers, A. *Canadian Journal of Emergency Medicine, 2011;13(5):310-318.* | **Methods:**  Paramedics (17) and EMS medical directors (7) across Canada, 24 participants.  Delphi study in four rounds. | **Aim:**  To achieve consensus among a group of Canadian EMS experts on the most important decisions paramedics make during typical high-acuity emergency calls in terms of clinical outcome and patient safety. | **Results:**  42 clinical decisions were determined as being important. Clinical decisions were sorted into the following decision categories:   - Airway management - Assessment - Cardiac management - Drug administration - General treatment - Scene management   Airway and cardiac management were considered most important for patient outcome and safety.  The majority of the decisions that have implication for clinical outcome and safety occur during the on-scene treatment phase.  **Conclusion:** | **Limitations:**  Methodological limitations of Delphi study. Decisions of what might be important may differ with another constitution of the panel.  The decisions selected as most important for patient safety and clinical outcome were defined by consensus and are not verified by actual patient outcomes or safety data. | Good |
| 10 | Ethical decision-making based on field assessment: The experiences of prehospital personnel | Torabi, M., Borhani, F., Abbaszadeh, A., & Atashzadeh-Shoorideh, F. *Nursing Ethics* | **Methods:**  Qualitative study using content analysis of semi structured in-depth interviews. Participants were 15 EMS staffs. | **Aim:**  To describe the experiences and identify the strategies used I EDM (ethical decision-making) by the prehospital EMS personnel in Iran. | **Results:**  Three major categories were constructed: ***Assessment of the scene atmosphere***   - The participants considered the patient’s perspectives and cultural values before carrying out any decision or medical intervention. Sometimes they tried to obtain information about the beliefs and cultural values of the region’s residents. - Some EMS personnel believed that a lack of cultural awareness could lead to negative consequences, such as complaints about the EMS personnel. - Time limits and unpredictability make EDM difficult and can affect ethical decisions. - The expectations of bystanders or patients may have an effect on the EMS personnel’s ethical decisions. This may be related to threats or “pushy” bystanders or relatives.   ***assessment of the patient’s condition and her/his family***   - The patient and disease characteristics influenced the decisions of the EMS personnel. If the patient was younger or had a family he/she was given more effort. - Family’s attitudes and wishes as well as their awareness and acceptance of the situation of the patient can affect EDM. EMS providers try to respect families’ opinions and their concerns.   ***predicting decision-making outcomes***   - If treatment seemed futile, particularly in CPR situations when there was a low chance of survival EMS personnel would restrain from CPR with the family’s approval. Factors affecting decision-making in EDM might come from the care provider or patient’s value system, religious and cultural context, healthcare professionals’ personal motives, and patients’ or family members’ requests and persistence. - Detecting potential risks or threats were an important factor in EMS personnel’s EDM. Ensuring their own safety against potential threats. Scene and job security (avoiding complaints regarding work effort from relatives of the patient in order to keep their job). - EMS personnel considered eventual legal consequences when making decisions about EDM.     A central category was also constructed, Field assessment, which demonstrated the strategy of EDM by prehospital providers when facing ethical conflicts.  **Conclusion:**  More research is needed regarding EDM and variables affecting it. | **Limitations:**  Explanation of the rigour throughout the study. | Fair |
| 11 | Factors influencing decision making among ambulance nurses in emergency care situations | Gunnarsson, B., & Warrén Stomberg, M. *International Emergency Nursing, 2009, 17: 83-89* | **Methods:**  A qualitative descriptive method. Interviews with 14 ambulance nurses in Sweden. | **Aim:**  To investigate which factors influence decision-making among ambulance nurses in emergency care situations | **Results:**  Five central categories were found:  ***The incident:***   - The extent of the incidents was of crucial importance for how the decisions were made. - The complex meetings with patients, relatives and other operators. - The severity of the incident, many injured, trauma cases, urgent states of ill-health, fires and taking care of patients in relation to serious crimes (murder) - Caring for intoxicated persons and addicts, partly from the perspective of their own safety, but also the difficulty interpreting the symptoms. - The complexity of the situation must always be seen in a context. - The patients’ physical and psychological status was a part of the decision-making basis. - Age was a significant factor, especially children and older patients. Taking care of children was the worst aspect of the job. Older patients sometimes made it easier to refrain from life-supporting treatment. - Ambulance nurses formed a first impression on the basis of what they saw. A matter of quickly getting an overall picture of the situation. - Initial examination was used for decision-making based on vital signs, awareness, respiration and circulation. Critical or non-critical conditions were assessed.   ***The external factors:***   - Time of day, the season and weather conditions were of significance. It was easier working in daylight and good weather. - Distance to the alarm address, as well as the state of the roads affected driving times. - The amount of time before further resources could arrive. - Stress could affect the decisions despite the participants’ knowledge. - Own security was a primary concern for the ambulance staff - Persons surrounding the injured or sick patient affected decisions. Relatives might be an aid but also constituted an obstacle in the work. The relatives’ impact on decisions was most clearly illustrated by the presence of the parents of injured children. - Expectations or perceived expectations of onlookers was considered as a factor influencing decision-making.   ***Communication and cooperation:***   - The initial information enabled ambulance nurses to prepare themselves mentally for what they might face at the scene. - Lack of information was also interpreted. - Previous knowledge of the personnel (fire rescue or police) clinicians worked or cooperated with was seen as a positive factor for collaborating. - Communication and cooperation with colleagues influenced decision-making. In teams who knew each other well the ambulance staff were able to use non-verbal communication. - Feeling safe and trusting a colleague’s judgement was important. - A functional leadership at the scene was important for decision-making. Being able to act as management and distribute the work on scene so as to give other staff members information and relevant duties.   ***Knowledge:***   - Education was seen as a prerequisite to making correct decisions. - Previous experiences of caring and monitoring patients’ progress, observing symptoms and changes in the patients’ condition over time was seen as a major advantage in the prehospital assessment of patients. - Ambulance staff need to make knowledge their own since they often work alone. - Making mistakes and reflecting over alternative actions led to new knowledge being created. - The participants often trusted their instincts and acted accordingly. They reflected upon and analyzed the event afterwards.   ***Ethical dilemmas:***   - A formally correct decision might feel wrong ethically. - Guidelines might be experienced as an obstacle to the work in certain contexts, like starting resuscitation when they felt it was meaningless. - Ambulance nurses often acted according to how they would want to be treated themselves in the same situation. - Patients’ integrity was sometimes a problem when working in their homes or with other people present. The risk exists of giving offence even if decisions are medically correct. - The more seriously injured the patient was, the less the patients’ own views were taken into account.   **Conclusion:**   - The extent of the incident and the degree of difficulty are decisive in how decisions are made. - Ambulance nurses’ experience is important for decision making. - External factors such as the uncertainty of a prehospital environment, expectations and pressures from an environment in which one is working while being observed by other people, insufficient information, leadership problems and collaboration with many different operators, all contribute to making decisions in an urgent situation even more complex. | **Limitations:**  There may have been another result if a more narrative kind of method had been chosen.  More male than female ambulance nurses participated. | Fair |
| 12 | Gaps between policy, protocols and practice: a qualitative study of the views and practice of emergency ambulance staff concerning the care of patients with non-urgent needs | Snooks, H A., Kearsley, N., Dale, J., Halter, M., Redhead, J., & Foster, J. *Quality & Safety in Health Care* *2005;14:251-257.* | **Methods:**  Two focus groups (one intervention and one control) for evaluation of how a service development was received, how frontline staff make decisions to convey or leave patients at the scene. How they felt about introduction to a new protocol and about using it.  Qualitative data with some quantitative measurements. A mixed method.  United Kingdom | **Aim:**  To report the views and attitudes of emergency ambulance staff concerning their current routine practice and the new intervention allowing them to make decisions to leave 999 patients at the scene of their call. | **Results:**  7 out of 10 invited from the intervention station participated in the initial focus group, 6 out of 10 in the follow up session. 8 out of 16 invited from the control station attended the pre-trial focus group, 5 out of 16 attended the follow-up session.  Factors influencing the participants’ decisions about non-conveyance were experience and intuition, pragmatism and patients/carers’ circumstances or attitudes.   - Conveyance was seen as the ‘easy option’. When in doubt the participants conveyed to A&E, mostly because they did not believe that they were not covered legally to leave a patient at the scene. - Operational circumstances influenced their decision. For example, if the shift had been difficult, busy with a lot of call outs to minor problems, or if it was near the end of their shift and they wanted to leave work on time, then conveyance to A&E was more likely. - Conveyance decisions were also influenced by the attitudes of patients/carers and their social situation. If they had social support or access to a district nurse or GP, it was more likely for the crews to be prepared to leave the patient at home. - Some participants thought that the new protocol gave a legitimacy to an informal practice that already occurred. The new protocol could improve their confidence and job satisfaction and make crews more consistent in their assessment and decision-making. - Some participants had concerns regarding the patients who did not fit neatly into the protocol. They felt that the protocol might leave crews open for criticism when using their own judgement. - Some participants felt lack of trust from their own service backing them up as well as for other healthcare providers to work with them to arrange self-care for the patient. - Concerns were raised about collaborating with other agencies, that they would not be available once the project started. - Crews felt that they would have needed more training in using the protocol and that they would have wanted more support from the ambulance service management and trainers. - Crews felt that they had gained clinical knowledge through their training in using the protocol. - Crews felt that there was benefit to the patients because the ambulance crews were more systematic in their assessments and had increased confidence in their decision-making. However, some participants felt that their practice was somewhat driven by their instincts rather than the protocol. - Referring patients to GPs and other services was still perceived as very problematic, both in making these arrangements but also to get the patients to accept other treatment or self-care instead of A&E.   **Conclusion:**  Ambulance crews were generally positive to the new model both before and after implementation of the intervention. There are some difficulties surrounding successful implementation. To change a practice and service delivery is complex, both within the ambulance service but also across boundaries with other service providers. | **Limitations:**  The study site was selected on the basis of the anticipated compliance of crews, the extent to which views expressed are representative of crews elsewhere is unknown. | Fair |
| 13 | Grey areas: New Zeeland ambulance personnel’s experiences of challenging resuscitation decision-making. | Anderson, N., Gott, M., & Slark, J. *International Emergency Nursing, 2017* | **Methods:**  Interviews with 16 ambulance personnel. Analyzed with an interpretive phenomenological analysis. | **Aim:**  To identify the clinical, ethical, cognitive and emotional challenges that emergency ambulance personnel experience when making decisions to commence, continue, withhold or terminate resuscitation. | **Results:**  Four over-arching themes captured challenging decision-making:  ***Grey areas – situations where key information was unavailable or conflicting***   - Incomplete knowledge of key background information about the patient and circumstances of arrest, or a mix of favourable and poor prognostic factors could make decision-making more challenging. - The participants needed an intrinsically determined amount of concordant verifiable information to withhold or terminate resuscitation.   ***Exceptional cases – first-encounters, arrests of secondary aetiology and those involving children or young people***   - Children or young people were rarely encountered but highly anticipated events which triggered emotional responses from crew and bystanders. In these cases resuscitation efforts were more often initiated and sometimes continued for a longer time than might have been associated with cardiac arrest in a much older person, even among the most experienced participants. - Secondary cardiac arrest was also associated with challenging elements. - Crew safety and uncertainty.   ***Scene challenges – including the expectations and responses of bystanders, limited resources or difficult patient access***   - Expectations and behaviour of bystanders. Unrealistic expectations of bystanders. The participants might need to adjust their actions and communication with each other, mindful that they had an audience. - Some participants described commencing or continuing resuscitation primarily or exclusively to ensure crew safety.   ***Personal responses – the idiosyncratic impact of individual values and emotional triggers***   - Participants’ personal values and emotional responses had a modulating influence on decision-making certainty. - Personal and professional experiences with death, grief, disability, stress and distress could have an impact on decisions, however the participants’ beliefs differed. - Differences in personal values meant participants did not always agree with the decisions of other ambulance personnel.   **Conclusion:**  Uncertainty and challenge were associated with a number of features, including the patient, scene, arrest aetiology and available information. Awareness of personal values and emotional responses appeared to have a modulating effect. | **Limitations:**  The results might not be transferable to other populations. A majority of participants had long experience of working in the ambulance services, which might be both positive and negative. However, all participants conceded that they continue to face challenges when making resuscitation decisions. | Fair |
| 14 | Managing uncertainty in paramedics’ decision making | Harenc ˇárová, H. *Journal of Cognitive Engineering and Decision making, 2017;11(1):42-62.* | **Methods:**  9 paramedics from Slovak urban areas were interviewed.  Critical decision-making was used combined with a mixed method. | **Aim:**  To identify the types of uncertainty paramedics experience and the strategies they use to manage uncertainty at different phases of the incident and provide a detailed description of what uncertainty in this domain means. | **Results:**   - The situation had a negligible impact on the type of uncertainty and type of strategy. The situation explained less than 0.0001% of the variance in uncertainty and strategy. This means that the situation itself has very little to do with uncertainty and strategy and that these themes were similar across the situations reported. - Initial uncertainty concerned the patient’s condition and how it might further evolve. - Uncertainty regarding action included decisions on how to treat the patient, what equipment to take from the ambulance and how to transport the patient. - Coping strategies in the initial phase were primarily reduction followed by forestalling. - Most cases of uncertainty regarding outcomes were solved by forestalling. - The strategy of weighing pros and cons was used only in cases of uncertainty regarding action. - In the first phase the paramedics dealt with uncertainty over the location of the patient, the safety of the environment and what equipment to take from the ambulance. - During the incident-handling phase the most cases of uncertainty were managed by reduction through active information search. Information could be gained from medical history and examination of the patient, and by asking patient, family or bystanders, or looking for documentation. - Forestalling was used in two cases: to secure the environment and to prevent possible patient deterioration. - Forestalling or prioritization were used when deciding on bringing equipment, transportation aids or if the patient was in an acute life-threatening situation (not bringing transportation aid, but both personnel and equipment). - Uncertainty regarding cause was mainly managed through reduction. - Mistakes made by a novice, as perceived by the participants, would be not communicating appropriately, omitting or not carrying out procedures or examinations thoroughly, handling equipment poorly, underestimating the patient’s condition and not asking for assistance.   **Conclusion:** | **Limitations:**  Further studies are needed to confirm these findings.  Retrospective studies always have their limitations. Methodological triangulation would be an option to allow the capture of the potential differences between methods. | Good |
| 15 | Out-of-hospital decision-making and factors influencing the regional distribution of injured patients in a trauma system. | Newgard, C., Nelson, M., Kampp, M., et al. *Journal of Trauma, 2011;70(6):1345-1353.* | **Methods:**  A mixed methods approach.  10 EMS agencies. Observation and interviews with 35 EMS field providers and a round-table discussion with 40 EMS management personnel.  Country - USA | **Aim:**  To evaluate how decisions are made in the out-of-hospital setting, EMS provider cognitive reasoning, reasons for selecting specific hospitals, and the resulting hospital distribution of injured patients in a well-established trauma system. | **Results:**   - Patient/family preference and proximity were the prevailing reasons for selecting certain hospitals among injured patients not entered into the trauma system. - EMS judgement was the most common reason for trauma activation, rather than specific triage criteria. - EMS personnel rationalize their decision-making processes based on experience and a culture that encourages conservative decision-making and accommodates over-triage. - The triage process was heavily influenced by the initial dispatch information, early visual cues from the scene, and provider experience. - The information gathered during the subsequent phase of patient assessment is generally used to support the already-made decision for trauma system activation, but not to refute it. - All participants recognized judgement to be the most common way of identifying seriously injured patients. - Several providers stated that triage algorithms were “for newbies” and generally not useful for more experienced field providers, though all providers supported the triage algorithms for provider education and developing a framework for trauma triage. - For patients not identified as seriously injured, EMS providers consistently stated patient/family preference to be the next most important factor for hospital selection. If they do not have a preference, the proximity to hospital is the main criteria. EMS personnel often tried to match the patients’ need to hospital resources. - There was little or no penalty for over-triage but under-triage is strongly discouraged and penalized on multiple levels.   **Conclusion:**  Trauma decision-making is rooted in provider intuition, experience, and early informative cues. | **Limitations:** | Fair |
| 16 | Paramedics’ experiences of end-of-life care decision making with regard to nursing home residents: an exploration of influential issues and factors | Murphy-Jones, G., & Timmons, S. *Emergency medicine Journal, 2016;33:722-726.* | **Methods:**  Semi-structured interviews with a phenomenological approach.  6 participants (3 men, 3 women).  Analysis through thematic approach.  England | **Aim:**  To explore how paramedics make decisions when asked to transport nursing home residents nearing the end of their lives. | **Results:**  Three themes emerged in relation to transporting patients from nursing homes to EDs in end-of-life care situations:  ***The challenges in understanding the patients’ wishes***   - The paramedics expressed wanting to understand the patients’ wishes and expected these to be documented. - Where documentation was available it was considered to be limited in content. Most often these wishes were restricted to resuscitation decisions. - Paramedics tried to used professional carers, relatives and friends to describe conversations where preferences might have been expressed.   ***Evaluating patients’ best interests***   - When patients’ were not considered to have the capacity for decision-making, paramedics described a desire to act in their best interests. - Factors used to evaluate a patient’s best interests were: their diagnosis, comorbidities, quality of life, wishes where known and current condition. - Half of the paramedics could not describe how they calculated patients’ best interests but all demonstrated a process of balancing the perceived risks and benefits of hospitalization. - Risks of conveying included: causing psychological harm (distress and disorientation), physical harm (discomfort), these were balanced against the perceived benefits of meeting their clinical needs, reducing suffering, providing reassurance and accessing care not available in the community.   ***The influence of others on decision-making***   - A paramedic’s decision about conveyance was subject to external influences from nursing home staff, patients’ relatives and other paramedics. - All paramedics described actively seeking involvement from patients’ relatives in decision-making but remained aware of the potential bias arising from relatives’ wishes. - Uncertainty in decision-making was evident, several participants sought confirmation from others about what actions to take. - Conveyance to hospital was seen to afford protection, remove responsibility and avoid potential complaints or legal proceedings.   **Conclusion:** | **Limitations:**  Small study sample, all recruited from one ambulance service.  Transferability is restricted due to recruitment at a single site. | Good |
| 17 | ‘Popping nana back into bed’ – a qualitative exploration of paramedic decision making when caring for older people who have fallen. | Simpson, P., Thomas, R., Bendal, J., Lord, B., Lord, S., & Close, J. *BMC Health Services Research, 2017;17:299(1-14).* | **Methods:**  A qualitative study design incorporating constructivist grounded theory methodology.  Setting: a large, state-based Australian ambulance service.  Semi-structured in-depth interviews and focus groups. Thematic coding and analysis. | **Aim:**  To investigate paramedic decision-making and develop a theory explaining paramedic decision-making whilst providing care to older people who have fallen. | **Results:**  In total, 33 paramedics participated, 13 in interviews and 20 in four different focus groups. Four theoretical constructs arose:  ***Role perception:***   - Participants experienced confusion over their role and this had a substantial impact on the decision-making process when caring for older fallers. - The confusion consisted of thoughts that EMS work is delivering emergency, life-saving care to patients with “legitimate” health problems. There appears to be a cultural determination that high acuity work involving patients with potentially life-threatening conditions is “legitimate” or “good work”. - Participants saw themselves as highly trained to manage patients with life-threatening conditions and it became evident that everything around them, culturally and organizationally, reinforces that this is what they are here to do. This however is not the reality of ambulance service provision. - The paramedic role was portrayed in media through stories about major motor vehicle accidents, helicopter rescues, major traumatic injuries and resuscitation for cardiac arrest situations. - The perception of “legitimate” work affects the approach to a case and the decision-making process that follows. - Despite the frustration arising from incidents of older fallers, the frustration was aimed at the ‘incident’ and not the patient. - A higher or more advanced education or training level was a factor for thinking that older fallers constitute ‘legitimate’ work. And they also had a more realistic expectation of what their work would consist of. - There is a risk that paramedics who disdain low acuity work will be complacent and have unhelpful preconceptions before arriving at the scene. There is a risk they will make poor decisions based on a cursory examination, poor information gathering, and sub-optimal clinical decisions. - Those who thought old fallers were ‘real’ cases tended to describe a hypothetico-deductive approach to decision-making and find a clinical challenge in falls cases. Those who felt that falls cases were not ‘real’ cases appeared to use a more pattern-based decision-making approach, anchored in unqualified experiences of past cases and heuristics.   ***Education and training:***   - Paramedics’ capacity to make good decisions is limited in part by the quality of education and training they receive. - Paramedics reported feeling ill-prepared to manage cases of older fallers. This was supported by paramedic educators who acknowledged that falls-related education and decision-making training were insufficient. - Most paramedics looked for and fixed injuries as a priority in the assessment process. Those with higher education levels tended to assess fall risk factors, or investigation of medical causes in the decision-making process. - Most paramedics thought that their organization did not think this kind of work important since they received little or no training in management of older fallers. However, an algorithmic falls protocol was introduced to support decision-making in these cases. Even if the protocol was introduced 2 years prior to the study the paramedics were generally unfamiliar with it. - Paramedics thought that the protocol was too constricting for paramedic practice.   ***Operational demands:***   - Paramedics felt constant pressure to be available to respond to ‘real’ work. This pressure may be cumulative, intensifying as they are repeatedly dispatched to perceived low-acuity work. - This stress can manifest itself in shortcut assessments that provide limited opportunity for holistic assessment of an older faller. - Pressure is promoted from dispatch and organization through behaviour and performance measure indicators promoting ‘restrictive’ scene times with no perceived consideration for the clinical needs of the patient.   ***Confidence:***   - Decision-making by paramedics when caring for older fallers is strongly influenced by a pervading fear of not being supported by their organization if an adverse event were to arise secondary to their decision-making. - Cynicism toward the organization was contextually mostly in relation to non-transported patients, a decision which many were uncomfortable making due to the perception that they were opening themselves up to risk of organizational reprisal. - Lack of confidence fuelled a strong culture of “cover your arse” in which decisions were frequently made based on what is best for the paramedic (paramedic-centred decision-making). - This approach (of transporting all older fallers) to managing transport decision, removes the need to make a decision. - Paramedics also lacked confidence in their own clinical ability to manage an older faller. - Paramedics who had confidence in themselves and their knowledge made decisions despite lack of trust in the organization.   **Conclusion:**  Paramedic decision-making when providing care to older people who have fallen, is profoundly influenced by perception of role and the cultural and organizational constructs of what is real work for paramedics. Perceived inadequacy of preparedness to provide care for older fallers, characterized by insufficient education and training, lack of confidence, and constant pressures arising from management of operational demand, inhibit the provision of patient-centred, carefully considered care for this population. | **Limitations:**  The study was conducted within a single ambulance service so the findings might not be generalizable to other populations of paramedics in other ambulance services.  The researcher is also a paramedic which may have led to bias in interpreting the data. | Good |
| 18 | Situation assessment and decision making strategies of emergency medical services physicians in routine and non-routine situations. | Sedlár, M. *Studia Psychologica, 2017;59(2):127-138.* | **Methods:**  15 ground EMS physicians, in Slovakia and the Czech Republic, two different companies.  Critical Decision Method was used. It is a retrospective semi-structured in-depth interview investigating critical or non-routine challenging situations from professionals’ own practice. | **Aim:**  To identify situation assessment and decision-making strategies in emergency medical services physicians and determine whether their distribution is associated with routine and non-routine situations. | **Results:**  There were 93 decision points involving Situation Assessment and 172 decision points involving Decision-making.   - Most decision points in routine and non-routine situations concerned the patient’s medical condition (88 related to SA, 164 related to DM). - Only in non-routine situations were the decision points also about the patient’s behavioural and psychological characteristics, physical environment, and other people’s behavioural and psychological characteristics. - The most frequently used strategy was intuitive SA, as it was called by the researcher due to difficulties of determining whether SA was developed by pattern-matching (seen as intuitive strategy) or feature-matching (used intuitively). - Decision points using deliberative feature-matching and mental simulation were only found in four non-routine situations and were related to the patient’s medical condition. - Participants used both previous experiences and medical knowledge to make sense of health problems that did not present themselves as suspected in order to reach some SA. - One to five hypotheses concerning diagnoses were generated and evaluated. - In non-routine situations about 85% of assessments were developed intuitively, and 15% deliberately, whereas in routine situations 100% of assessments were developed intuitively. - Intuitive DM was the most frequently used strategy in non-routine situations (83%) as well as in routine situations (93%). - Intuitive decisions were accompanied by the automatic implementation of the first experience-based option that came to mind. - Deliberate DM strategies were used in three routine situations and in 12 non-routine situations, in which decision points were related to various situational factors. - Only in non-routine situations, did the participants mentally simulate (3%) hypothetical consequences of each identified option and evaluate their plausibility in terms of risks for the patient. - Analytic DM appeared less in routine situations (7%), in contrast with non-routine situations (11%). - Only in non-routine situations did the participants ask for decision justification by asking for advice from the EMS team member. - Decisions might be based on advice/recommendation from other professionals (dispatch operator, fireman, or physician in the specialized healthcare centre or a request from the patient’s relative. - In non-routine situations 83% of decisions were made intuitively and 17% deliberately, whereas in routine situations about 93% of decisions were made intuitively and 7% deliberately. | **Limitations:**  A small sample size and retrospective subjective data, the results should be taken carefully. The study does however verifies some aspects of the recognition primed decision making model in two types of situations. | Fair |
| 19 | The prehospital assessment of severe trauma patients’ performed by the specialist ambulance nurse in Sweden – a phenomenographic study | Abelsson, A., & Lindwall, L. *Emergency Medicine, 2012;20(67):1-8.* | **Methods:**  Phenomenographic design in which 15 specialist ambulance nurses from varying parts of two counties in Sweden participated.  Data collection through interview | **Aim:**  To describe specialist ambulance nurses’ perceptions of assessing patients exposed to severe trauma. | **Results:**  Structural themes were:  ***To be prepared for emergency situations:***   - Focused on the ability to assess emergency situations and on evaluating and inquiring for confirmation about prior experience. - Being able to create an image of the surroundings and of the influencing factors of the trauma upon arrival at the scene was considered important. - Finding out what had happened at the time of the injury in order to interpret the amount of violence the body had been exposed to and how the energy of the impact had injured the patient. - The ability to read the scene of the accident was based on theoretical knowledge from the specialist education and from experience of having seen accidents and patients exposed to trauma before. - Previous hospital experience of working with trauma patients added to the participants’ knowledge of differential diagnosis. - Since assessment of trauma patients rarely occurred the participants did not obtain proficiency, this meant that they were ready to act but did not feel safe in the situation. - Theoretical knowledge and practical experience provided a sense of flexibility in the assessment. - The overview of the patient and the situation was described as a feeling of knowing whether a patient was in critical condition or not. - A reflective mind-set provided an opportunity to take a step back and reassess patients who were not responding to the treatment as expected. - Experience resulted in humility and confidence in the fact that not everything always goes as expected. It is possible to be wrong and have the courage to say it out loud. - Experience also led to an avoidance of tunnel vision and not being paralyzed, which opens up for more information being processed simultaneously. - Confirmation of whether the assessment was right or wrong is essential. Feedback provided knowledge and experience for the next event. - Discussion with an experienced specialist ambulance nurse was used as a control mechanism and confirmation of the approach used. By evaluating that one’s own actions were consistent with those of others enabled a sense of confirmation was reached. - Subsequent talks with emergency room staff aimed at knowing the patient’s injuries which helped improve the treatment of patients with similar injuries. - Guidelines were supportive of the assessment and facilitated the management of responsibility. However, the guidelines were sometimes experienced as restrictive or not sufficiently clear, because prehospital environments differ more than is predictable. - Rapid decisions taken at the incident site were not always supported by guidelines, which subsequently could be challenged by the receiving hospital.   ***Confidence in one’s own leadership:***   - The leadership responsibility of the safety for the EMS personnel and patient care as the priority was considered a big responsibility. - If the participants had the opportunity to prepare mentally before arriving at the incident there was an improved quality of work. - Working with competent staff made delegation quick and smooth. - Previous experiences of working at the scene of an accident helped when handling stress. - The education of specialized ambulance nurses was appropriate for the clinical work and made the participants more structured in their assessments.   ***Developing professional knowledge:***   - The specialist ambulance nurses described working with personnel from other specialties, such as intensive care and nurse anaesthetists, when it came to discussions and reflections on physiology and medicine. However, these had no experience of prehospital work and practical work in the ambulance service. Even doctors were considered to provide hospital-adapted care and therefore fell short in prehospital settings. - Being a mentor and teacher for those with a lower level of education and experience was described by the participants as creating a safer working environment for all of them. - Being alone at the incident site was described as a shortcoming when a lot needed to be done. There was a sense of security that emerged in the work with colleagues with the same specialist education. - Having a colleague with the same specialization was described as important, a feeling of being equal, opposed to having a non-registered nurse as assistant. It reduced the risk of getting stuck in a single pattern of thought or tasks being forgotten. - Being familiar with the equipment was described as important. - Seeking new knowledge through reading scientific literature and articles could maintain and update the knowledge. - A sense of uncertainty was raised about not being able to handle the situation, but still being expected to know everything. | **Limitations:**  Phenomenology is a description of the specific participants’ thought and how they see the world.  The differences in the prehospital care setting in Sweden compared to those in other countries. Specialized ambulance nurses are trained to work independently in prehospital care, being responsible for assessment and treatment of patients. | Fair |
| 20 | Termination of prehospital resuscitative efforts: a study of documentation on ethical consideration at the scene | Mikkelsen, S., Schaffalitzky de Muckadell, C., Grassmé Binderup, L., Lossius, H M., Toft, P., & Touborg Lassen, A. *Scandinavian Journal of Trauma, Resuscitation and Emergency Medicine, 2017:25;35:1-9.* | **Methods:**  A retrospective study based on the documentation from the MECU registry and the MECU discharge summaries. All patients in whom the decision to allow for natural death was made were included. Study period Jan 2010 to December 2014.  Denmark | **Aim:**  To investigate to what extent ethical considerations are documented in discharge summaries in cases of life-and-death decisions made by emergency care anaesthesiologists in a Danish prehospital setting. Furthermore, the study aims to describe the nature of such considerations and seeks to argue for the establishment of recommendations for documentation of ethical considerations in discharge summaries. | **Results:**  The MECU made the crucial decision to resuscitate or to allow natural death in 1,275 patients. In 642 of these patients, resuscitative efforts were initiated at the scene. In 633 patients, natural death was allowed without any resuscitative efforts being initiated.   - Only 62 patients (4.9%) had medical records containing specific ethical or philosophical considerations pertaining to the event. In these 62 patients, a total of 85 individual observations of ethical considerations were made. - In 36 of 633 patients for whom resuscitation was not attempted a DNR-order was presented. - In 21 patients the main considerations influencing the physician not to initiate treatment was the patients’ expected quality of life after the incident. - In 17 patients the physician’s reason for refraining from treatment was the patient’s estimated life expectancy following a hypothetically successful resuscitation attempt. - In 6 patients the reasons for not initiating any resuscitation attempts but to allow natural death to happen was the knowledge of end-of-life wishes or the expectations of the patients or the relatives. - DNR-orders were sometimes overruled by the patient’s relatives insisting on treatment. - An infant found in its cot received attempted resuscitation even though the physician documented that it was considered obviously futile, out of consideration for the parents who were not ready to accept that their child was dead. - If resuscitation was started by bystanders or relatives the physician might feel obligated to continue with resuscitation attempts, especially if ROSC had been achieved. - There was no documentation of additional ethical concerns, for instance concern for the interest of the physician, EMT, nursing staff or chance-passers-by, and issues of prioritization of resources in the larger perspective of society.   **Conclusions:**  An improved practice of documenting ethical considerations in pre-hospital life-and-death decisions is needed. A template should be implemented in prehospital medical records describing the basis for any decision being made. | **Limitations:**  A thorough review of all medical record and databases pertaining to each individual mission. Three authors independently registered the information.  Only addresses patients cared for by one organization, which might reflect the documentation culture of that single institution. | Good |
| 21 | Prehospital trauma triage decision-making: A model of what happens between the 9-1-1 call and the hospital | Jones, CMC., Cushman, J., Lerner, Brooke., Fisher, S., Seplaki, C., Veazie, P., Wasserman, E., Dozier, A., & Shah, M. *Prehospital Emergency Care, 2016;20:6-14.* | **Methods:**  A two-stage qualitative study involving EMS providers. Four initial focus groups, and five additional confirmatory focus groups.  USA | **Aim:**  To model the decision process and to identify potential assessment factors related to the decision to transport to a trauma centre. | **Results:**  Eight themes were identified: rapid evaluation, use of estimation, provider intuition, provider education/training, thought process, protocol application, patient factors, and system factors. These eight themes coalesced into four overarching domains:   - ***Initial assessment:*** upon arriving at the scene the providers make an initial assessment within the first minute and this drives their ultimate choice in destination hospital - EMS providers trust their initial instincts and “listen to their gut”. - The general appearance of the patient upon arrival is more valuable than a more detailed assessment and precise measurement of vital signs. - The initial “scene size-up” drives the subsequent evaluations and assessments the providers make. - ***Importance of speed versus accuracy:*** *EMS providers placed significant emphasis on the rapidity of their assessments and decisions.* - Providers often reported using “estimates” or dichotomous normal/abnormal impressions, rather than specific numeric measurements, to inform their choice of destination hospital. - Precise measurements are often taken during transport to validate the providers’ impressions. - “Estimates” of vital signs such as blood pressure or Glasgow Coma Scale were inferred based on general impressions of patients using on-scene simple interactions, rather than taking precise measurements. - ***Usability of current field triage criteria:*** Guidelines in a stepwise fashion for trauma triage do not correlate with how the EMS providers actually make their assessment in the prehospital setting. - Providers stated that trauma triage is not a linear process that involves step-by-step evaluations, in contrast to the current structure of the FTDS. - The selection of receiving hospital begins while driving to the scene based on dispatch information, and is further informed by their initial assessment upon scene arrival. - The patient severity upon arrival at the scene dictates what assessments the providers will make and in what order. - The mechanism of injury and obvious anatomic injuries are assessed first, followed by impression of the patient. - Taking vital signs is often not feasible upon initial scene arrival and may delay care. They are often taken to validate observations and the impression of EMS provider. - The participants rely more on general patient impression than specific numbers because the specific values outlined in their triage guidelines do not account for the baseline status of patients. - ***Consideration of patient and emergency care system-level factors:*** Participants described that in cases in which an immediate decision cannot be made, other factors, such as patient preference, trauma centre proximity, available resources at non-trauma centres, and “busyness” of the trauma centre are vital in their triage decisions. - Patient preferences are often incorporated into trauma triage decisions whenever possible, particular in the rural settings. - Rural EMS providers expressed concerns regarding the lengthy transportation time to the closest trauma centre. - Calling for back-up or interception is often required in cases of severe trauma for providers in rural areas. - Participants working in rural areas remarked that stabilizing the patient and transporting the patient to the closest hospital is often weighted against making a 1-hour or longer drive to the trauma centre. - Rural participants were also more concerned about the availability of helicopter transport in poor weather conditions than urban/suburban participants.   **Conclusion:**  Speed is essential to the EMS providers’ decision-making process and destination decisions are often made based on information that is available immediately upon scene arrival and/or their initial impression. For patients who are not immediately recognized as requiring trauma centre transport, a secondary and usually more detailed evaluation of other factors is made. Rural providers carried out a risk-benefit analysis, weighing system factors against the patient’s need for trauma centre care. | **Limitations:**  Participants were from one geographic area and one EMS system. The authors tried to mitigate this through purposefully sampling a diverse range of advanced and basic life-support providers from urban/suburban and rural EMS agencies. | Fair |
| 22 | Refraining from pre-hospital airway management: a prospective observational study of critical decision making in an anaesthesiologist-staffed pre-hospital critical care service | Rognås, L., Hansen, T M., Kirkegaard, H., & Tonnesen, E. *Scandinavian Journal of Trauma and Resuscitation Emergency Medicine, 2013;21:75.* | **Methods:**  Prospective observational study.  1081 cases  Denmark | **Aim:**  To study the critical decision-making process associated with the decision not to perform PHAAM. | **Results:**   - The most common reason for withholding PHAAM in the study system is patient condition and patient co-morbidity. - PHAAM was sometimes not performed due to a short transportation distance to hospital. - Other reasons for withholding PHAAM were associated with too high risks of complications or cases where the anaesthesiologist deemed PHAAM futile or unethical. | **Limitations:**  The participants registered their data which might lead to registration bias or recall bias. However, a high capture rate reduces the risk of selection bias. | Good |
| 23 | We are strangers walking into their life-changing event: How prehospital providers manage emergency calls at the end of life. | Waldrop, D., Clemency, B., Lindstrom, H., & Clemency Cordes, C. *Journal of Pain and Symptom Management, 2015;50(7):328-334.* | **Methods:**  An exploratory, descriptive and cross-sectional study design.  178 prehospital providers had completed a self-administered survey.  43 participants agreed to be interviewed. | **Aim:**  To explore and describe how prehospital providers assess and manage emergency calls near death. | **Results:**  Four themes emerged that illustrated the dynamics of emergency end-of-life calls:   - ***Multifocal assessment:*** the participants described the importance of rapidly and simultaneously assessing the patient, family, and environment on arrival. - Patient assessment involved the identification of the medical issues (cardiac arrest, vital signs, diagnosis and comorbid conditions, if the event was witnessed, and time elapsed). - Family assessment involved identifying relationships between the patient and others present and whether there was a surrogate decision-maker, a do-not-resuscitate order, and advance directives. - The participants also assessed the interactions between family members for emotional intensity and volatility. - Environmental assessment involved scanning the household and bystanders to assess the safety of the scene. - The family’s intense emotion and distress were described as potentially making the environment unpredictable. - Environmental assessment was described as observing the physical and interpersonal atmosphere and incorporating it into the intervention. - Assessment of the family members’ level of awareness and comprehension of the diagnosis, coexisting conditions, medications, and the dying process was described as central. - A pervasive lack of understanding about 911 calls, ambulance transport, and ED care was described. - ***Family responses:*** Assessments of emotional and behavioural responses were described as key to determining subsequent interventions. - Emotional desperation that can accompany imminent death as the demand for providers to do something. The request for action was described as fulfilling an emotional need although the family often may not be sure what they specifically want the providers to do. - Concerns about safety can be intensified when providers are in the confined and unfamiliar environment of a patient’s home. - ***Conflicts:*** When patients have a completed DNR order but the document cannot be located, prehospital providers are legally bound to start resuscitation even if a family member states that the person does not want it. - Sometimes patients have a completed DNR and lack capacity, some families ask prehospital providers to resuscitate in the crisis of a looming death. - Conflicts arise from when patients do not want to be transported to a hospital but the family members do. - ***Management of the dying process:*** Management of family responses and conflicts during the dying process influences decision-making in the field. Participants noted that their interactions with family members were often more difficult than the patient care when death has occurred or is imminent. - Sometimes participants transported the patient to the hospital when the family was intensely reactive as means to stabilize a crisis.   **Conclusion:**  The importance of managing symptom crises and stress responses that accompany the dying process is particularly germane to quality care at life’s end. These results suggest the importance of increasing prehospital providers’ abilities to uphold advance directives and patients’ end-of-life wishes while managing the emotionality of the event and supporting families. | **Limitations:**  Participants were drawn from a single EMS agency and were primarily Caucasian males. A more diverse sample may provide more varied responses. | Fair |
| 24 | Paramedic practice – Knowledge invested in action | Wyatt, A. *Journal of Emergency Primary Health Care, 2003;1(3-4);* | **Methods:**  Ethnographic techniques through a case-study using observation and interviews. | **Aim:**  To examine the utilization of tacit knowledge by experienced paramedics made explicit through their application of professional judgement | **Results:**   - **Role of Experience:** All participants confirmed that their experience was a major contributor to their knowledge base and their ability to make sound clinical decisions. - Experience in isolation is not the answer to developing expertise. It is available to be drawn on, and this is indeed valuable, however it was suggested there are other factors involved. - Experience may be seen as a reflective tool when it was used to guide judgements and provide an avenue for recognition of difference as well as similarities. - Experience was primarily used in cases that appeared to be routine. - Less routine cases often called for a more openly combined effort in the management of the patient, leading to discussion between paramedics where they were open to sharing their experiences in an effort to arrive at a suitable course of action. - The acquisition of experiential knowledge was seen to be broad-based, and the sources of such knowledge diverse. Even life experiences external to the workplace environment. - Observation of other paramedics operating within the workplace context was important as well as gaining broader information from patients and their relatives. - There is an inability to capture adequately the nature of the knowledge gained, highlighting again the tacit nature of expert judgements and the difficulty in making these processes explicit. - **Learning within a specific context:** Paramedics mainly gained knowledge within the specific context of their workplace, but also recognized the importance of a strong general knowledge base. - When the paramedics were novice they adhered more rigidly to protocols and struggled to make difficult judgements. - **The expert paramedic’s clinical approach:** Expert paramedics describe not taking all the “steps” described by a textbook in their assessment of the patient but instead identifying important clinical issues more quickly and focusing on these. - Expert paramedics were reluctant to label patients with a specific diagnosis, but rather looked at the problem in a more holistic sense. This made them more open to a variety of causes for the patient’s clinical presentation. - The expert practitioner seemed to display a form of reflectiveness in their approach to patients and the problems they present. This affords the expert practitioner the opportunity to consider multiple means and multiple ends to clinical problems and not be directed by established rules and guidelines. - **Making judgements:** it was important to be able to perform several tasks concurrently, perhaps without having all information available to them at the time. - With experience comes a development of confidence in making judgements – the capacity to act anticipatively. - To be able to learn from both good and bad experiences. - **Conclusion:** | **Limitations:** | Fair |
| 25 | Are prehospital treatment or conveyance decisions affected by an ambulance crew’s ability to access a patient’s health information? | Zorab, O., Robinson, M., & Endacott, R. *BMC Emergency Medicine, 2015;15:1-7* | **Methods:**  Extensive review of literature provided (created) an online, two-part questionnaire. | **Aim:**  To   - identify how ambulance clinicians currently access health information and barriers that prevent crews accessing data. - ascertain whether a lack of information could lead to a suboptimal care pathway being selected. - explore whether, in hypothetical scenarios, increasing the amount of information available would lead to selection of a more appropriate care pathway | **Results:**  302 clinicians completed the first part, and 285 completed the entire survey.   - 94,4 % of respondents felt that they had been unable to access health information about a patient that they were caring for, - whilst 90.7 % felt that this lack of information had led to a less appropriate care pathway being selected. - Information that was not available which could have helped them make a decision, 86,8 % of respondents recalled that a patient’s past medical history was not readily available - 77.2% were unable to gain prompt access to a patient’s resuscitation status or end-of-life care wishes. - The main source for information on a patient’s medical history was though a GP (94.7 %). - Respondents who entered their current job through higher education were more likely to use additional sources of information. - A longer length of time working in the ambulance service had an impact on usefulness of information about the patient’s normal vital signs, and also the use of Treatment Escalation Plan information to inform the conveyance decision.   **Conclusion:**  Accurate health information is vital to make safe conveyance decisions and a lack of access could result in patients being unnecessarily conveyed to an emergency department when alternative care pathways may be appropriate and available.  Up-to-date health information and records are the best source for making safe clinical decisions. | **Limitations:**  Only looked at one regional ambulance service and may not be representative of practice experienced in other parts of the UK.  A low response rate (12 %). | Good |
| 26 | Caring assessment in the Swedish ambulance services relieves suffering and enables safe decisions | Wireklint Sundström, B., & Dahlberg, K. *International Emergency Nursing, 2011;19:113-119* | **Methods:**  A reflective lifeworld approach within the healthcare science context.  Data collection was through participant observation, field notes and interviews. | **Aim:**  To describe and illuminate pre-hospital emergency care with particular emphasis on assessment.  How can professional carers´ assessments be understood? What characterises assessments made by experienced carers in the ambulance services? How do the carers approach their patients? | **Results:**  ***Making room for caring assessment:***   - The pre-hospital scene is unplanned and each care situation has to be arranged in relation to the circumstances. - Carers need to make room for care, both symbolically and manifestly. - Carers take control of a situation that is unstructured and perhaps confusing, and asserting such control is particularly important when the pre-hospital environment is a public setting. - Carers express ambitions to generate feelings of calm and peace when dealing with the patient and to facilitate the assessment.   ***Being close to the patient:***   - In order to accomplish assessments and provide appropriate care, carers ensure close contact with the patient. - The patient’s condition is continuously assessed through dialogue, eye contact and touching, while maintaining focus on the technology.   ***Care governed by the patient’s lifeworld and suffering:***   - Since the carers in pre-hospital care often care for only one patient at a time the carer can proceed more slowly and get to know the patient in order to assess and understand her/his suffering. - Carers take an interest in the patient’s perspective in order to ensure that correct and necessary actions are undertaken and conversely avoid unnecessary interventions.   ***When care is governed by the patient’s medical condition:***   - Carers make use of time in a medically effective manner, where assessments and interventions follow standardized formats. - Following a medical model, the assessment is characterized by the fact that one intervention is quickly followed by the next. Carers can be fully occupied or even absorbed by monitoring the medical equipment and measurements and consequently not pay appropriate attention to the person they are caring for.   ***Co-operation benefits the assessment:***   - Assessment benefits from the interaction between carers who complement each other when caring for a patient. Such interactions include verbal as well as non-verbal communication. | **Limitations:**  Only one author did the data collection. However, the second author asked critical questions or saw other meanings in the material.  Ordinary limitations of phenomenological studies in regard to their inability to capture completely the participants’ lived experiences or feelings. Which can influence the results. | Good |
| 27 | View from the door: Making pediatric transport decisions based on first impressions. | Mierek, C., Nacca, N., Scott, JM., et al. *JEMS, 2010;35(7):68-9* | **Methods:**  Ethnographic analysis strategies for development of themes.  12 EMTs observe two videos of paediatric patients and make a transport decision based on their observations.  USA | **Aim:**  To determine if experienced providers can use the information gathered from the “view from the door” to make transport decisions on paediatric patients, and if that information fits with the PAT. | **Results:**   - In 92 % the participants verbalized a clear, simultaneous airway/breathing assessment. The method for assessment was observation of anatomical deformity, chest rise, crying (or lack of), spitting, coughing, belly breathing, head bobbing, respiratory rate, accessory muscle use, retractions, head shaking, audible respiration, congestion, respiratory effort, head position, ability to speak and paradoxical breathing. - In 75 % the participants verbalized an assessment of the patient’s level of consciousness. The method for assessing this was general interaction with surroundings, playing with a toy, body position, and posture, crying, following commands, answering questions, moving all limbs and showing a lack of fear. - In 58 % the participants verbalized an assessment of the patient’s circulatory status. The method for accessing this was observation of skin colour, blood loss, contusion to the abdomen, palmar pallor, and cheek pallor or peri-oral cyanosis. - In 50 % the participants verbalized a treatment plan. This often included a justification for stay and play or load and go. Treatment plans included treatment during transportation strategies, such as supplemental oxygen and IV-fluids. - In 21 % participants verbalized an anticipation of a poor outcome, mostly concerned about the patient going into shock.   **Conclusion:**  A transport decision was arrived at in less than 20 seconds. During this time providers were formulating treatment plans and anticipating potential negative outcomes. | **Limitations:**  A small sample size. | Fair |
| 28 | Ambulance nursing assessment: part two | Bruce, K., Dahlberg, K., Suserud, B-O. *Emergency nurse; London, 2003 (2013); 11(1):14-18.* | **Methods:**  Phenomenology. Interview with 6 ambulance nurses. The participants prepared a description of when their judgement had been decisive for the patient.  Sweden | **Aim:**  To examine nurses’ progress in pre-hospital emergency care from first alarm until the completion of nursing measures. | **Results:**   - When ambulance nurses are confident of the medicine involved, the way is open for proper nursing relationships. - Trust is the key to meaningful relationships. And the immediate contact is crucial to whether nursing care can even begin. - The nature of the contact between any nurse and patient is determined by the patient’s needs. - Conscious patients should be communicated with directly. - In unconscious patients, communication is established indirectly, with someone who can describe the patient’s case. - Nurses must, out of respect for patient integrity, show empathy and learn to share the patients’ feelings. - Patients’ body language may reflect their suffering. - Nurses can register situations in their entirety and then decide what must be done first. - Nurses can obtain consent to carry out nursing duties first after trust has been achieved. - Patients’ surroundings are important when making assessment because social circumstances and home environment reflect patients’ daily lives and enhance an overall picture. - Nurses can show respect for patient integrity by respecting patient culture. - Nurses’ plans at pick-up point are followed by primary assessment - Primary and secondary assessments follow a set pattern in terms of vital functions. - Secondary assessment confirms initial plans so they can be implemented or allows them to be reconsidered. - Some cases require distance in relations between nurses and patients so that thoughts and feelings can be subordinated to the work.   **Conclusion:**  Close contact with patients has great importance in situations where nurses are responsible for emergency care. Nurses rely on experience to reveal patient needs, which are complex in such circumstances and can be physical, emotional, social and cultural in character. Another factor when making assessment is the possibility of ambulance nurses being the only nurses on the scene. Which makes judgement in an already complicated situation even more difficult. | **Limitations:**  Small sample size.  Does not mention any limitations in discussion. | Fair-Poor |
| 29 | Ethics in treatment decisions during out-of-hospital resuscitation | Naess, A-C., Steen, E., & Steen, P. A. *Resusciation, 1997;33:245-256* | **Methods:**  In-depth interview with 9 doctors and 35 paramedics who were interviewed in pairs.  Norway | **Aim:**  To elicit the criteria actually used by paramedics in the Oslo EMS system when making decisions about CPR. Also to find out whether these criteria tended to differ from the criteria used by doctors on the physician-manned ambulance and if they were affected by their length of experience. | **Results**  There was a similar and limited set of criteria used by both the paramedics and doctors when deciding whether to discontinue CPR in the field or to continue CPR during transport.   - Some signs that indicated a good prognosis always led to start of CPR by paramedics and doctors. - All stated that they always started resuscitation in cases of confirmed ventricular fibrillation. - Other factors that always led to initiation of CPR were patient gasps, or small apparent movements of the jaw, constricted pupils or abnormal (IS THIS WHAT YOU MEAN? MM) skin colour. - Time intervals, either estimated collapse-to-first CPR attempt, or known dispatch-response interval were mentioned. - If unsure the participants usually started CPR and then asked relatives about the time interval. - Old age was never a criterion for not starting CPR although young age was a criterion for starting it. However old age in combination with comorbidities were a reason for discontinuing CPR. - If a bystander started CPR, the participants always continued in order to give positive feedback in starting it, regardless of whether the bystander’s CPR seemed effective. - Expectations expressed by the relatives or bystanders affected the participants’ decision to start CPR. - The participants sometimes found the relatives to be an emotional burden in the emergency situation - The relatives’ expectations could also influence the decision to abstain from or to cease CPR. - Sometimes a patient was transported to the hospital with ongoing CPR to avoid facing relatives with the information that CPR was futile. - If the relatives had accepted the patient’s death upon arrival of the participants they would probably not start CPR. - Threats from friends or relatives were a reason for starting CPR. These threats mostly came from people under the influence of alcohol or drugs. In a few cases they were emphasized by a knife or a gun. - The reputation of the EMS system was an important factor in starting or not starting CPR, depending on what they perceived to be the relatives’ expectations - Some participants thought that the decision to start or not start CPR was a great responsibility and also a burden. - Previous experience was reported as a criterion in the decision-making. - There sometimes the need for CPR practice (?) - Although not mentioned as a reason for deciding whether or not to start CPR, some participants said that they were aware that the social status of the patient could influence their decision and claimed that they had given much thought to the problem of negative influence. | **Limitations** | Fair |
| 30 | Exploring factors affecting emergency medical services staffs’ decision about transporting medical patients to medical facilities. | Ebrahimian, A., Seyedin, H., Jamshidi-Orak, R., & Masoumi, G. *Emergency Medicine International, 2014; id215329, 1-8* | **Methods:**  Qualitative content analysis approach.  All EMS staffs working in prehospital care facilities in the north, eastern, western and southern districts of Tehran, Iran.  18 EMS staffs participated. | **Aim:**  To explore factors affecting EMS staffs’ decisions about transporting medical patients to medical facilities.   - How do you identify that a medical patient needs transportation? - What do medical patients and their families expect from you? - How do medical patients and their families react to your decisions about patient transportation to medical facilities? - What are your criteria for transportation? | **Results:**  ***Patients’ condition:***   - The most important criterion affecting the participants’ decisions for transportation was the patients’ condition. - The presence of a serious disease or obvious acute signs and symptoms directly helps the participants make transport decisions. - Also, patients’ healthy physical state as well as the absence of a serious disease made participants suspicious about a real need for transportation. - Socioeconomic status was a factor contributing to transportation decisions. - Patients’ support system (legal, family, neighbours and insurance coverage), affected the participants’ decisions about transportation. - Legal support included health-related rules and regulations that in most cases safeguard patients’ not care providers’ rights. - Families obliged the participants, either kindly or forcefully, to transport their patient. - Health insurance coverage was also an important factor contribution to families’ insistence upon transportation. - EMS staffs’ judgement about family members’ ability to re-call for ambulance and successfully deliver care to their patient until ambulance arrives were also a factor. - Educational status of patient and his family was a factor that both facilitated and hindered the participant’s ability to make a right decision about patient transportation. - A higher educational status as well as having health-related education facilitated patients’ and their families’ understanding of EMS staffs’ duties, encouraged them to provide staffs with accurate information about patients’ conditions, and helped them respect staffs’ decisions. - In some instances, patients and family members who had higher educational status tended to hold higher expectations. - Patients and family members who held health-related degrees, sometimes meddled in EMS staffs’ affairs and negatively affected their decisions. - Patients and family members who had low educational status, factors such as misunderstandings and misconceptions about EMS staffs’ duties, inability to establish effective communication, inability to establish effective communications with staffs, inability to recall patient’s medical history, and indifference towards the importance of keeping patient’s medical records made decisions regarding transportation difficult. - Patients and families with higher financial status held higher expectations and showed higher sensitivities to their health. - Patients and families with lower financial status were more willing to receive care at home. - Patients and families that trusted the EMS staffs’ abilities were more cooperative, provided more accurate information and respected the EMS staffs’ views and decisions. - Lack of confidence in the EMS staff might result in reluctance to provide information about past medical history and present illness. - The patient’s and family’s beliefs and attitudes towards EMS services and their staff contributed to their tendency to abide by EMS staffs’ decisions. These beliefs and attitudes were often based on previous first- or second-hand experiences of EMS efficiency and safety. - Characteristics of the mission were a factor. Although most of the missions follow a consistent pattern, the conditions and characteristics of each mission are unique and differ from other missions. - Response time was a factor. Late arrival, irrespective of its cause presented EMS staff with decision-making difficulties about transportation, through the unfavourable emotional atmosphere of emergency situations. EMS staff usually decided on transportation of patients to alleviate the condition and lighten the atmosphere. - The possibility of obtaining advice was a factor for decision of transportation. - Equipment was a major factor contributing to transportation decision. The inaccessibility or defectiveness of essential equipment made EMS staff suspicious about assessment data and therefor compelled them to transportation. - Special cases, such as elderly living alone, students in school, culprits and prisoners, and foreigners were almost always transported to medical facilities irrespective of the severity or the seriousness of the problem, because EMS staff were afraid of being accused of negligence or malpractice. - EMS staffs’ ability to reason developed over time through gaining knowledge and experience which also could affect their decisions regarding transportation. - Medical problems were harder than obvious injuries and traumas to diagnose. - EMS staffs with more knowledge and experience have better reasoning ability and hence reach sensible decisions more easily and more quickly. - The physical health status of EMS staffs affected their transportation decisions. (Fatigue, sleepiness, the flu, headache and musculoskeletal pain) - The strength of EMS staff’s support system, including legal, organizational, professional, managerial, and financial support as well as liability insurance coverage was also a key factor affecting the participants’ decisions about patient transportation. EMS that did not have an effective support system made decisions that carried minimal risk. | **Limitations:**  Participants might have taken into account different personal and organizational considerations when sharing their experiences even though the authors strove to create a comfortable and supportive environment during the interviews. | Fair |
| 31 | Factors influencing hospital transport of patients in continuing cardiac arrest | Hick, J., Mahoney, B., & Lappe, M. *Annals of Emergency Medicine, 1998;32(1):19-25* | **Methods:**  Prospective data collection of study sheets that paramedics attending unsuccessful resuscitation completed.  USA | **Aim:**  To determine the factors that influence transport of victims of out-of-hospital continuing cardiac arrest and to define problems with field termination of resuscitation efforts. | **Results:**  In all 259 cardiac arrests were attended during the data collection period. In 79 of the cases the patient was pronounced dead on the basis of clinical criteria. 180 patients were resuscitated and transported to hospital. 68 patients were transported while in continuing cardiac arrest.   - Paramedics made the decision to transport in 54 of the cases, physicians did so in 14 cases. - The single greatest reason for transportation was cardiac arrest in public places. - Medical factors were another main reason for transportation. - Possible correctable causes (overdose, carbon monoxide poisoning, choking and PEA). - Persistent refractory VT/VF prompted transport in some cases. - Inability to start an iv-line and airway problems were indication for transportation. Because ACLS had not yet been possible. - Cultural barriers were cited in one case in which a hysterical, non-English-speaking family was present but no interpreter was available. - In two cases family explicitly rejected field termination. In another the paramedics believed that the family was too unstable to accept field termination. - Extreme obesity. - Communication failure, not being able to get hold of additional resources or medical advice. | **Limitations:**  The paramedics may have noted “physician ordered transport” in some cases to avoid listing factors influencing transport, or did not think carefully about their reasons for initiating transport.  The influence of past practice to transport may influence some groups of paramedics more than others.  The physicians’ reasons for transportation decisions is unavailable.  The data collection was mainly done in the winter period which makes hypothermia a reason for transportation. | Poor |
| 32 | How is the diagnosis made? The observation of paramedics performance in simulated completion task | Gurnáková, J., & Harencárová, H. *International conference on Naturalistic decision making, 2013, Marseille, France.*  Czechia | **Methods:**  (Video)Observation of 28 paramedic teams in selected tasks was compared with objective medical evaluation of their performance. | **Aim:**  To assess the frequency and nature of deviations from a standardized treatment protocol of paramedic teams in the selected competition task.  COMPETITION or COMPLETION? See “How is the diagnosis made?” | **Results:**  ***Processing of initial information from emergency dispatch centre:***   - Further information, other than given from dispatch, processing slightly correlated with higher evaluation of approach towards the patient from the patient himself. - There was some tendency of more successful teams to study and process information in the instruction more thoroughly. - Reading aloud, while simultaneously selecting key information about the patient, formed an appropriate image of the situation on the spot and also selected the best possibilities for further more probable treatment of the patient. - Interaction with team colleague regarding the instruction enabled them to correct the primary misunderstandings. - The least successful strategy was not paying full attention to the instructions, reading them on the go, ignoring the possibilities of patient treatment, inadequate understanding of situation or fixation on the anamnestic detail (he was speaking strangely).   ***Anamnesis and examination:***   - An ideal solution of the task was careful and systematic taking of the anamnesis, thorough examination: physical examination, measuring non-invasive values, establishing glycaemia, 12-lead ECG and complete basic neurological examination. - Even the most successful teams did not perform the task without mistakes. - Teams with a narrow focus just on application of their examination procedure often overlooked the important anamnestic information spontaneously provided by the patient. These teams also forgot to ask about possible connections with actual state of patient’s health. - Some teams listened actively to the patient, relied completely on his information and did not verify his state by examination. - None of the teams did a complete examination. - Successful procedure in this phase of dealing with the task was systematic examination: head to toe, with simultaneous taking of the anamnesis, perceiving the relevant information spontaneously obtained from the patient and its verification by examinations. - It was helpful to summarize the information gained after some intervals and its verification, an effort to understand the situation. - An alternative was also a combination of the mostly systematic procedure of anamnesis-taking with a parallel examination, while at the same time verifying the preliminary hypotheses, perceiving information from the patient and deriving new plausible hypotheses, but only if the paramedic was still able to return to the systematic procedure.   ***Diagnosis:***   - Although 23 (of 28) teams came to the right conclusion that the patient was healthy, this conclusion was not well supported by the examinations conducted. - Some teams showed nervousness, uncertainty or helplessness, as the number of examinations with good results increased. - Two teams had a tendency to stick to any kind of minor discrepancy in the examination results and use it as an argument for the next examination by the doctor. - Two teams refused to accept the idea that the patient was healthy despite all good results and they concluded that the patient had to be intoxicated or a psychiatric case. They presented this as a reason for forced transport. | **Limitations** | Fair |
| 33 | Paramedic clinical decision-making: results of two Canadian studies | Jensen, J. *International Journal of Paramedic Practice, 2011;* | **Methods:**  Combining the results of two different studies in Canada.  8 participants. | **Aim:**  Focus on paramedic clinical decision-making, specifically, the judgements paramedics make that pertain to assessment, treatment and transport decisions.  Which clinical decisions paramedics make are most important for patient safety and clinical outcome, and how paramedics make clinical decisions. | **Results:**   - The ability to be able to “fit” medical procedures into the paramedic work context is an important part of paramedic work. - Decisions regarding airway management and cardiac management were considered most important of the clinical decisions. - The on-scene treatment phase of the typical emergency call has the highest decision density. - Type 1 (dual process) thinking is more prone to error, it is effective in minimizing reaction time, avoiding “paralysis by analysis”. - Type 2 (dual process) thinking is used in more complex situations where paramedics weigh pros and cons of each option and make a conscious decision on the best fit. - Type 2 by proxy refers to paramedics using “thinking tools” to speed decision-making. (algorithms, clinical prediction tools, and differential diagnosis lists) - In trauma scenarios paramedics used event-driven and algorithmic thinking most frequently. - In medical scenarios paramedics used algorithmic or “rule out worst scenario” thinking. - Novice paramedics failed to verbalize their decisions more than experienced paramedics. - No differences in thinking strategies were found between novice and experienced paramedics. - Some health professionals do not believe that algorithms and clinical prediction rules can out-perform clinical judgement and may feel these are a threat to their decision-making autonomy and lead to inflexible care plans. - Several reviews comparing clinical judgement to clinical prediction rules found that these tools are almost always more accurate and often require less clinical information than individual clinician judgement. - In a chaotic or time-sensitive situation, it is preferable for paramedics to use decision tools, rather than rely on Type 1 (dual process) processes, which can be influenced by bias, the emotional state of the thinker, and inappropriate use of mental short cuts. - It is also preferable to taking too much time deliberating each competing hypothesis or decision option using Type 2 (dual process) processes before acting. | **Limitations** | Poor |
| 34 | Performance of Experienced versus less experienced paramedics in managing challenging scenarios: a cognitive task analysis study | Smith, M W., Bentley, M A., Fernandez, A R., Gibson, G., Schweikhart, S B., & Woods, D D. *Annals of Emergency Medicine, 2013 (???)* | **Methods:**  Cognitive task analysis.  2 scenarios.  10 paramedics (6 experienced and 4 less experienced)  USA | **Aim:**  To explore the performance of more experienced paramedics through the use of scenarios involving sense-making challenges and diagnostic reasoning in the context of care management and resource and task management challenges, identification of leverage points, use of resources, compared to less experienced paramedics. | **Results:**   - Less experienced tended to give more focused assessments (myocardial infarction in chest pain) while the more experienced tended to be broader in their assessments. - More experienced paramedics addressed transportations and trying to stabilize the patient while still continuing to explore possibilities until they found out the cause. - More experienced paramedics considered more hypotheses than less experienced. And the hypotheses were more specific than the ones of the less experienced. - More experienced paramedics made more assessments, particularly pulmonary assessments and taking history. - More experienced paramedics were more likely to notice negative results from an intervention and incorporate them into hypothesis revisions. - Most paramedics did not become constrained to only one hypothesis, there were some instances in which discordant evidence did not trigger a revision. - Paramedics in both groups provided adequate care and transportation despite the constraints imposed in the difficult scenarios. - Less experienced paramedics were either thorough with one patient or conducted basics for both patients (multiple patient case). - More experienced paramedics were able to balance their focus between two patients (multiple patient case) - More experienced paramedics, to a higher degree (3/4) made an assessment in anticipation of a possible tension pneumothorax compared with less experienced paramedics (1/4). - More experienced paramedics made additional assessments to a higher degree than less experienced paramedic during the cases until handoff. - More experienced paramedics used their EMT in a greater variety of ways than did less experienced. | **Limitations:**  A relatively small sample size, although not atypical for this kind of study.  The simulation did not incorporate social factors such as familiarity between members in an established ambulance crew.  Only a small set of complexity factors was incorporated. | Fair |
| 35 | Reasoning processes used by paramedics to solve clinical problems | Alexander, M. *Diss.* *2010.* | **Methods:**  9 participants  USA | **Aim:**  What are the cognitive processes by which paramedics solve a clinical problem in a given patient scenario?   - How do paramedics identify and interpret the meaning of clinical information in the problem-solving process? - What problem-solving strategies do paramedics use to arrive at a provisional diagnosis when solving clinical problems? - What hypotheses or differential diagnoses are developed to explain the patient’s problem? - What reasoning strategies are used by paramedics to develop a plan to address the patient’s problem? | **Results:**  ***Scenario 1***   - Participants developed between 1 and 7 hypotheses (mean 3.3) about the patient’s problem. - Most of the participant (8/10) followed the patient’s chief complaint for assessment. - Overall participants used relatively few attributes to develop, rule in, and rule out hypotheses, although additional attributes were often available. - Some participants, in hindsight, stated that they left out important attributes related to the history and physical examination. However, a few participants (2/10) thought that they had a clear picture of the patients’ main issue. - Of the five participants who obtained and interpreted a 12-lead ECG, none interpreted it similarly. And only one was consistent with the interpretation provided by an instructor in advanced cardiology. - The presence of risk factors for a specific disease or pathophysiologic precursors was a frequently elicited attribute used either in developing or supporting the hypothesis. - Pseudo-information sometimes persisted despite the patient’s denial of it (E.g. Patient did not complain of chest pain, yet chest pain was generated by several participants even though the patient only complained about difficulty breathing and palpitations). - Inductive and deductive reasoning, algorithms, stereotyped approaches, and mnemonics played prominent roles in the collection of attributes subsequent to determining the patient’s chief complaint. - When algorithms or stereotyped approaches were used, participants were not always sure why the information was collected, or what meaning it had. - Inductive processes were commonly used (identification of attributes and then forming hypotheses). - Participants did not always follow their mnemonic (SAMPLE) to obtain patient’s medical history. They could ask for some of the components, although not necessarily in sequence or at a single specific point in the scenario. The same goes for OPQRST. - The preferred approach in conscious, stable medical patients, was a focused history and focused examination based on the patient’s chief complaint   ***Scenario 2***   - Participants generated between 1 and 5 hypotheses (mean 3.3). - Participants used relatively few attributes to develop, rule in, and rule out hypotheses, even though additional attributes were often available. - In some cases, no medical history was obtained, and no physical examination was performed. - Pseudo-information - SAMPLE was used. - However a common mnemonic used to guide the search for causes of altered mental status was not explicitly used by any of the participants even though alcohol, overdose and diabetic emergencies were commonly discussed by participants. - Both inductive and deductive reasoning were used to process attributes to search for or support hypotheses, to determine of justify treatment, and to make decisions regarding diagnoses. - All participants ultimately provided the basic support of airway, breathing, and circulation required for general management of patients with altered mental status. - 6/10 participants administrated Naloxone even though there were no signs of drug intoxication. - Anchoring seemed to be an issue with most of the participants. | **Limitations:** | Fair |
| 36 | The impact of stress on paramedic performance during simulated critical events | LeBlanc, V R., Regehr, C., Tavares, W., Scott, A K., MacDonald, R., King, K. *Prehospital and Disaster Medicine; 2012;27(4):369-374* | **Methods:**  22 advanced care paramedics.  Simulation scenarios.  Testing of cortisol levels and scoring on checklist and global rating scales.  Canada | **Aim:**  To examine paramedics’ stress responses and performance during simulated stressful clinical cases | **Results:**  - Anxiety levels were higher at the end of the scenarios than at baseline.  - High-stress scenarios increased anxiety more than a low-stress scenario.  - Cortisol levels increased during high-stress scenarios.  - Scores on the global rating scale were significantly lower in the high-stress scenario than in low-stress scenario.  - The paramedics committed more errors of commission (reporting information that was not present in the scenario) following the high-stress scenario.  - There were no differences in the number of omission errors (failing to recall information that was present in the scenario) between the low- and high-stress scenarios.  - The errors of commission in the high-stress scenarios consisted of reporting procedures or symptoms (e.g. administration of acetylsalicylic acid or jugular vein distention) which did not occur during the scenario, and of reporting the results of assessments (e.g. abdomen soft and non-tender, changes in pain rating after nitroglycerine, absence of dizziness) that were not made during scenarios. | **Limitations:**  Are the findings generalizable to clinical events with real patients?  However, some stressors that might not be in a real-world case might appear in simulation. | Fair |
| 37 | The Space-Control Theory of Paramedic Scene-Management | Campeau, A. G. *Symbolic Interaction* 2008;31(3):285-302 | **Methods:**  Grounded theory. 24 participants (paramedics) were interviewed in three rounds.  Canada | **Aim:**  To understand the dynamic aspect of paramedic practice – the process of scene management. With a differencing between novice and expert paramedics. | **Results:**   - Paramedics do not have predetermined work areas but accept the location where the patient is found and adapt themselves and the surrounding environment accordingly. - The adaptation is directed toward enabling patient care. - Paramedics achieve this adaptation by controlling activities taking place in the surrounding environment. This includes physical and human elements. - The scene management is a dynamic, social activity composed of social processes. - Interaction with other people is a part of scene management. - The paramedics need to shift focus on various elements, sometimes they are in the background and sometimes in the foreground. But still always present and determining outcomes. - There is a public perception that it is only the technical patient care procedures that comprise the important aspects of ambulance work. - Paramedic adaptation of the emergency scene includes five key theoretical categories of social processes: 1) establishing a safety zone, 2) reducing uncertainty through social relations, 3) controlling the trajectory of the scene, 4) dealing with temporality at the scene, and 5) collateral monitoring. - Each category contains a number of subcategories. Establishing a safety zone involves 1) the what-if strategy, 2) rationalized self-interest, and 3) trading off patient care and scene safety. - The category of reducing uncertainty through social relations involves 1) negotiating the division of labour with allied personnel, the inclusion of fire department personnel, and the exclusion of police personnel and 2) allocating tasks to non-allied personnel and reading the crowd, including assessing potential for assisting or hindering. - The category of controlling the trajectory of the scene involves 1) clinical considerations, 2) nonclinical considerations, 3) optimizing efficiency, including multitasking, and 4) modifying procedures. - The category of dealing with temporality at the scene involves 1) determining clinical urgency, 2) uncertainty of diagnosis and prognosis, 3) management oversight, and 4) scene circumstances. - The category of collateral monitoring involves 1) trading off attention and 2) optimizing efficiency. | **Limitations:**  None mentioned in article. | Fair |
| 38 | Non-conveyance of patients: Challenges to decision-making in emergency care | Leikkola, P. K., Mikkola, R. K., Salminen-Toumaala, M. H & Paacilainen, E.E.M. *Clinical Nursing Studies* 2016;4(4):31-39 | **Methods:**  Qualitative and descriptive. An electronic questionnaire was sent to 142 care providers, 71 replied.  Finland, September-October 2014.  Analysis through inductive content analysis | **Aim:**  To generate new knowledge of decision-making in emergency care situations, to be used in continuing vocational or professional education and in developing care quality.  Three research questions:   1. How difficult was it for care providers to make a decision on non-conveyance 2. What were the care providers’ reasons for not transporting patients to hospital? 3. Which factors related to non-conveyance did the care providers find challenging after the immediate emergency care situation? | **Results:**   - Reasons for not conveying were based on assessments of care needs and the conclusion that the patient did not require emergency treatment or transport to hospital. - Providers might advise the patient and relatives that they could go by their own vehicles or have family members accompany them. - Postponing the treatment a few hours seemed better than a longer nighttime transport by ambulance. - Patients seemed to expect that the ambulance would always convey them to hospital. - The patient received adequate treatment on scene. - If the patients’ complaint was not acute or had not deteriorated significantly, no emergency treatment was required. - Psychosocial reasons were the patient feeling lonely or needing someone to talk to. - It was also suggested that a lack of common sense or basic life skills sometimes resulted in unnecessary calls. - Some patients do not want to be conveyed. - There may be difficulties reaching a mutual understanding of non-conveyance, depending on different opinions of the patient’s needs. - Some thought that they would be treated more rapidly if conveyed by an ambulance and not needing to queue in the emergency clinic. - Care providers reported having felt pressurized to transport the patient. - Confusion, intoxicants or aggressive patients were harder to reach a mutual understanding with. - The care providers worried about the patient’s coping at home. - There was insecurity regarding the decisions of non-conveyance and the responsibilities that come with them. - Did the patient understand the advice of home care or would another call be made soon? - Does the patient receive adequate care from help services? - Unclear or ambiguous symptoms caused insecurity. - The care providers worried about making the wrong decisions. If insecure they would convey the patient to hospital. - The cooperation between professionals was considered challenging. | **Limitations:**  No limitations mentioned in article. | Fair |
